# BRCC3 mediates inflammation and pyroptosis in cerebral ischemia/reperfusion injury by activating the NLRP6 inflammasome

**DOI:** 10.1111/cns.14697

**Published:** 2024-03-28

**Authors:** Xiaohuan Huang, Junyi Tan, Yanyan Ji, Jing Luo, Yong Zhao, Jing Zhao

**Affiliations:** ^1^ Department of Pathology Chongqing Medical University Chongqing China; ^2^ Department of Pathology Chongqing Three Gorges Medical College Wanzhou China; ^3^ Department of Pathophysiology Chongqing Medical University Chongqing China; ^4^ Department of Neurology The First Affiliated Hospital of Chongqing Medical University Chongqing China

**Keywords:** BRCC3, cerebral I/R injury, neuroinflammation, NLRP6 inflammasome, pyroptosis

## Abstract

**Aims:**

Neuroinflammation and pyroptosis are key mediators of cerebral ischemia/reperfusion (I/R) injury‐induced pathogenic cascades. BRCC3, the human homolog of BRCC36, is implicated in neurological disorders and plays a crucial role in neuroinflammation and pyroptosis. However, its effects and potential mechanisms in cerebral I/R injury in mice are unclear.

**Methods:**

Cellular localization of BRCC3 and the interaction between BRCC3 and NLRP6 were assessed. Middle cerebral artery occlusion/reperfusion (MCAO) and oxygen–glucose deprivation/reoxygenation (OGD/R) models were established in mice and HT22 cells, respectively, to simulate cerebral I/R injury in vivo and in vitro.

**Results:**

BRCC3 protein expression peaked 24 h after MCAO and OGD/R. BRCC3 knockdown reduced the inflammation and pyroptosis caused by cerebral I/R injury and ameliorated neurological deficits in mice after MCAO. The effects of BRCC3 on inflammation and pyroptosis may be mediated by NLRP6 inflammasome activation. Moreover, both BRCC3 and its N‐ and C‐terminals interacted with NLRP6, and both BRCC3 and its terminals reduced NLRP6 ubiquitination. Additionally, BRCC3 affected the interaction between NLRP6 and ASC, which may be related to inflammasome activation.

**Conclusion:**

BRCC3 shows promise as a novel target to enhance neurological recovery and attenuate the inflammatory responses and pyroptosis caused by NLRP6 activation in cerebral I/R injury.

## Graphical abstract

Huang et al. confirm that BRCC3 plays a crucial role in the activation of NLRP6 inflammasome and inflammasome‐associated inflammatory response and pyroptosis in cerebral ischemia/reperfusion injury.

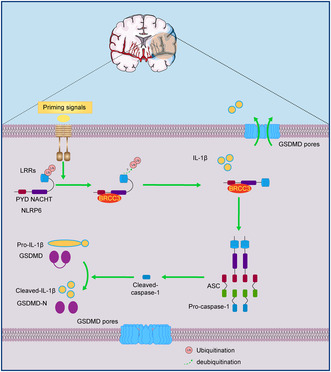

## 1 INTRODUCTION

Stroke is a cerebrovascular disease resulting in localized or overall brain tissue damage characterized by high morbidity, disability, and mortality.^1^ Stroke can be divided into ischemic, hemorrhagic, and subarachnoid stroke.^2^ The neuroinflammatory response is an important pathological process in early brain injury.^3^, ^4^ Activation of astrocytes and microglia and extravasation of leukocytes are the basis of the inflammatory response in the central nervous system. Activating the inflammatory response results in the release of large amounts of inflammatory factors, which promote the development of secondary brain injury.^5^

As a part of innate immunity, the NLR family^6^, ^7^ can recognize pathogen‐associated molecular patterns and damage‐associated molecular patterns in the cytoplasm after tissue damage or cell death and is part of the pattern recognition receptor family involved in inflammasome formation.^8^, ^9^ Multiple NLR family members regulate inflammatory responses in the central nervous system. For example, NLRP6 is closely associated with cerebral ischemia/reperfusion (I/R) injury^10^ and is involved in inflammasome formation, inflammation regulation, and defense against microorganisms.^11^, ^12^, ^13^ Upon activation, NLRP6 assembles with adapter ASC and effector pro‐caspase‐1 to form an inflammasome and further mediates caspase‐1 activation.^14^, ^15^ Activated caspase‐1 cleaves the pro‐inflammatory cytokines IL‐1β and IL‐18 into their mature forms through proteolysis and mediates the cleavage and activation of membrane pore‐forming protein gasdermin D (GSDMD), leading to the release of cytosolic contents, including the mature forms of IL‐1β and IL‐18,^16^, ^17^ which trigger pyroptosis. However, the specific mechanism underlying NLRP6 activation in cerebral I/R injury requires further investigation.

BRCC3, the human homolog of BRCC36, specifically recognizes and hydrolyzes ubiquitin chains formed via K63 links, reducing substrate ubiquitination modification levels; thus, it is also called ubiquitin hydrolase.^18^ BRCC3 can activate inflammasomes to mediate the inflammatory response.^19^ BRCC3 activates the NLRP6 inflammasome in rats.^20^ However, the effect of BRCC3 in deubiquitinating NLRP6 and its specific mechanism of action in activating NLRP6 during cerebral I/R injury in mice remain poorly understood. Therefore, investigating these aspects will provide deeper insights into the involvement of BRCC3 in neuroinflammation and pyroptosis, potentially paving the way for novel therapeutic strategies to mitigate the harmful effects of cerebral I/R injury and improve the neurological outcomes in patients.

We aimed to explore the effect of BRCC3 in neuroinflammation and pyroptosis after cerebral I/R injury in mice and the potential mechanism via which BRCC3 activates the NLRP6 inflammasome.

## 2 METHODS

### 2.1 Animals and cell culture

All experimental protocols and procedures were approved by the Ethical Committee of Chongqing Medical University, following the guidelines of the Experimental Animal Institute of Chongqing Medical University. A total of 153 male C57BL/6J mice (Chongqing Medical Animal Experimentation Center) weighing 18–22 g were used. All animals were housed under a 12‐h light–dark cycle at 21–26°C with unrestricted access to food and water.

Dr. Ling Deng provided the hippocampal neuronal (HT22) cell line as a gift. The HT22 cells were maintained in Dulbecco's modified Eagle's medium (DMEM) (C11995500BT, Gibco, USA) with 10% fetal bovine serum (FBS, Biological Industries, Israel) and 1% penicillin/streptomycin (Beyotime, Shanghai, China) in 5% CO_2_ at 37°C.

### 2.2 MCAO model

The MCAO surgical procedure was performed as previously described.^21^ The mice were intubated and kept under 3% isoflurane anesthesia (v/v in air). An incision was made at the neck midline under supine position. A nylon monofilament (AL1800; Jialing, Guangzhou, China) was inserted into the middle cerebral artery via the left external carotid artery for 1 h. The nylon monofilaments were withdrawn, and reperfusion was performed for 24 h. The Sham group was anesthetized, and the common carotid artery was located and sutured without embolization.

### 2.3 Laser Doppler flowmeter

The changes in cerebral blood flow were monitored using a laser Doppler flowmeter (LDF) (PeriFlux System 5000; Perimed, China) during MCAO modeling. We fixed the anesthetized mice to the locator, exposed the coronal and sagittal sutures, and used the intersection of the two sutures as the coordinate origin. We selected 1 mm to the left and 0.3 mm to the back for positioning. We detected a stable blood flow using an LDF after fixing the probe in place and before inserting the embolus into the internal carotid artery. The modeling was considered successful if the LDF decreased to 70%.

### 2.4 Cerebral infarct volume evaluation

To determine the volume of cerebral infarction, the entire brains of the mice were removed and kept at −20°C for 30 min before being sliced at 2 mm intervals (5 slices) as previously reported.^22^ The frozen sections were submerged in 2% 2,3,5‐triphenyltetrazolium chloride (TTC) (37°C; 15 min); this step necessitates protection from light. The samples were then fixed in 4% paraformaldehyde at 4°C for a period of 48 h. Images were obtained utilizing a digital camera and analyzed with ImageJ analysis software. Infarct volume was calculated as follows: {[total lesion volume − (ipsilateral hemisphere volume–contralateral hemisphere volume)]/contralateral hemisphere volume} × 100%.

### 2.5 Assessment of the neurological score

According to the literature, nerve injuries were scored blindly 24 h after modeling^23^ as follows: 0, normal; 1, inability to fully extend the contralateral forepaw; 2, turning to the contralateral side during locomotion; 3, tilted to the contralateral side; 4, inability to locomote spontaneously; 5, dead. Mice with a score of 0 and 5 were excluded.

### 2.6 Hematoxylin–eosin (HE) and Nissl staining

According to a previous study,^24^ after MCAO, the brains were rapidly collected and preserved in 4% paraformaldehyde for roughly 24 h. Next, coronal brain tissue sections, 2 mm thick, were cut from the whole brains and dehydrated. The sections were embedded in paraffin wax, stained with HE and Nissl, and observed under a microscope. Nissl‐stained neurons were quantitatively analyzed utilizing ImageJ software.

### 2.7 Oxygen and glucose deprivation/reoxygenation treatment of HT22 cells

After cell recovery, HT22 cells were grown to a 70%–80% density, trypsinized, passaged, and seeded in 10‐cm‐diameter culture dishes. Oxygen and glucose deprivation/reoxidation (OGD/R)^25^ experimental cells were placed in DMEM without glucose and cultured for 4 h in a Tri‐Gas incubator containing 1% O_2_, 94% N_2_, and 5% CO_2_. Glucose‐free DMEM was then replaced with a normal high‐glucose culture medium (DMEM containing 1% penicillin/streptomycin and 10% FBS), and the cells were cultured in a 5% CO_2_ incubator for 24 h. The cells in the control group were cultured normally without treatment.

### 2.8 Experimental design

Animals and cells were grouped as shown in the Supplementary Material S1.

#### 2.8.1 Experiment 1

Western blotting was used to determine the time course of endogenous BRCC3 expression in the left cerebral cortex. Immunofluorescence staining was performed 24 h after MCAO to determine the cellular localization of BRCC3.

#### 2.8.2 Experiment 2

To assess the effects of BRCC3 on neuroinflammation and pyroptosis, we knocked down BRCC3 using siRNA in mouse brain tissue 24 h before MCAO. We established 24 h cerebral I/R models in mice for detection. The grouping was as follows: Sham, MCAO, MCAO+NC, and MCAO+si‐BRCC3.

#### 2.8.3 Experiment 3

The time course of endogenous BRCC3 in HT22 cells was detected using western blotting analysis.

#### 2.8.4 Experiment 4

To elucidate the role of BRCC3 in neuroinflammation and pyroptosis, BRCC3 siRNA dissolved in diethyl pyrocarbonate (DEPC) was transfected into HT22 cells 24 h before OGD/R. To detect related markers, we established 24 h oxygen–glucose‐deprivation/reoxygenate.

#### 2.8.5 Experiment 5

To assess whether BRCC3 affects downstream inflammation and pyroptosis through NLRP6, a lentiviral‐packaged BRCC3‐overexpression plasmid was transfected into HT22 cells before OGD/R. To detect related markers, we established 24 h oxygen–glucose‐deprivation/reoxygenation.

### 2.9 BRCC3‐siRNA and NLRP6‐siRNA

We designed three BRCC3 siRNA mouse fragments: BRCC3‐mus‐1 (forward: 5'‐CCGGGUACUCUAUACUUGCUUTT‐3′ and reverse: 5'‐AAGCAAGUAUAGAG UACCCGGTT‐3′), BRCC3‐mus‐2 (forward: 5'‐CCCACCCUCAUAUAACUGUUU TT‐3′ and reverse: 5'‐AAACAGUUAUAUGAGGGUGGGTT‐3′), BRCC3‐mus‐3 (forward: 5'‐GCUCAGUAUUUACCAAGAAUUTT‐3′ and reverse: 5'‐AAUUC UUGGUAAAUACUGAGCTT‐3′), and the negative Control (forward: 5‐UUCUCC GAACGUGUCACGUTT‐3′ and reverse: 5'‐ACGUGACACGUUCGGAGAATT‐3′).

Firstly, we dissolved siRNA in DEPC water. After the mice were completely anesthetized, they were fixed on a stereotaxic device. After determining the location of the Bregma point, the mice were injected via the injection point. The position of the first injection point A was 0.3 mm behind the Bregma point, 1.0 mm beside the central axis, and 2.5 mm below the skull surface (coordinates: *x* = 0.3, *y* = 1.0, and *z* = 2.5). The coordinates of the second and third injection points were B (*x* = 0.5, *y* = 1.3, and *z* = 2.2), and C (*x* = 0.3, *y* = 2.2, and *z* = 2.2), respectively. The injection volume was 2.5 μL. The injection was performed over 5 min. After 10 min, the needle was slowly removed.

We inoculated the HT22 cells into 6‐well plates at a density of 1.5 × 10^5^/mL, 24 h before transfection. Before adding the transfection complex, HT22 cells were provided with a fresh culture medium. The transfection complex (Opti‐MEM; 125 μL, BRCC3‐siRNA; 5 μL, and Lipo8000™; 5 μL) was gently mixed, reacted at room temperature for 20 min, and then 125 μL mixture was added to each well. All were thoroughly mixed and placed into an incubator set at 37°C. OGD/R was performed after 24 h.

We designed three NLRP6 siRNA mouse fragments: NLRP6‐mus‐1752 (forward: 5'‐GGAGCUACGUGGUCAUCUUTT‐3′ and reverse: 5'‐AAGAUGACCACGUAG CUCCTT‐3′), NLRP6‐mus‐2643 (forward: 5'‐GCACCCUAAAUGCUCCCUATT‐3′ and reverse: 5'‐UAGGGAGCAUUUAGGGUGCTT‐3′), NLRP6‐mus‐1509 (forward: 5'‐GGAUCAUCAUAAAGCACAATT‐3′ and reverse: 5'‐UUGUGCU UUAUGAUGAUCCTT‐3′), and the negative Control (forward: 5'‐UUCUCCGAACG UGUCACGUTT‐3′ and reverse: 5'‐ACGUGACACGUUCGGAGAATT‐3′).

Once the density of BRCC3‐overexpressing HT22 cells reached 60%–80%, we transfected the NLRP6 siRNA, achieving efficient knockdown, and the OGD/R model was established 24 h later.

### 2.10 Western blot analysis

Equal amounts and volumes of protein samples (around 20–50 μg) were added into sodium dodecyl‐sulfate polyacrylamide gel electrophoresis (SDS‐PAGE) gel wells. Firstly, we used different voltages to perform electrophoresis of the proteins based on the gels’ protein concentration and separation ability. Electrophoresis was stopped when the bromophenol blue dye had migrated three‐quarters of the way through the SDS‐PAGE gel. Subsequently, the proteins were transferred to a polyvinylidene difluoride (PVDF) membrane (Millipore, Billerica, MA, USA) and incubated for 1 h at room temperature with 5% bovine serum albumin.

Following washing with TBST, the membrane was incubated with the corresponding primary antibody in an incubation box (4°C). All primary antibodies used are listed below: anti‐BRCC3 (1:200, Santa Cruz, USA); anti‐NLRP6 (1:2000, ABclonal, China); anti‐caspase‐1 (1:1000, ABclonal); anti‐IL‐1β (1:1000, Cell signaling, USA); anti‐GSDMD (1:1000, ABclonal); anti‐Flag (1:1000, Cell signaling); anti‐Myc (1:5000, ABclonal); anti‐HA (1:1000, Cell signaling); and anti‐β‐actin (1:10000, Abways, China). The membranes were incubated with the appropriate secondary antibodies (1:5000; Abbkine, USA) for 1–2 h at room temperature. Blot bands were detected using ECL (NCM Biotech, China). Lastly, we analyzed the grayscale values of the protein bands semi‐quantitatively using ImageLab version 6.0.

### 2.11 Immunofluorescence staining

The mice were deeply anesthetized 24 h after MCAO and transcardially perfused with 0.9% saline and 4% paraformaldehyde. Whole brains were removed and fixed in 4% paraformaldehyde for 24 h. The brain sections were incubated with primary antibodies overnight at 4°C. The primary antibodies used are listed below: anti‐BRCC3 (1:30, Santa Cruz, USA); anti‐neuronal nuclei (NeuN, 1:200, Abcam, USA); anti‐glial fibrillary acidic protein (GFAP, 1:200, ABclonal); anti‐ionized calcium‐binding adaptor molecule 1 (Iba‐1, 1:200, ABclonal); and anti‐NLRP6 (1:200, Bioss, China). Sections were washed with PBST and incubated with a secondary antibody for 1 h. Finally, the sections were stained with DAPI for 3–5 min at room temperature, and images of the samples were taken using a fluorescence microscope.

### 2.12 Plasmid construction and transfection

Primer designs are shown in Supplementary Material S2A.

Through primer design, gene amplification, enzyme digestion, ligation, transformation, bacterial selection, shaking, plasmid extraction, and sequence identification, the following plasmids were obtained: plenti6‐BRCC3‐myc_−_ddk, pcDNA3.1‐Myc‐NLRP6, pcDNA3.0‐3xFlag‐BRCC3, pcDNA3.0‐3xFlag‐BRCC3‐N, and pcDNA3.0‐3xFlag‐BRCC3‐C. The HEK293T cells were transfected when the density reached 70%–80%. We added Opti‐MEM (100 times the total mass of plasmid) and plasmid DNA (Myc‐NLRP6, Flag‐BRCC3, Flag‐BRCC3‐N, and Flag‐BRCC3‐C 6 μg), left the mixture to stand at room temperature for 5 min, and then added polyethylenimine (PEI) (4 times the total mass of plasmid) in proportion, mixed, and incubated the mixture at room temperature for 20 min. The HEK293T cells were passaged in a 1:1 ratio (one dish was transferred to another). Finally, the transfection complexes were mixed thoroughly into the cells. The cells were collected 48 h after incubation.

### 2.13 Construction of overexpressed plasmid and screening of stable transmissible cell lines

Primer designs are shown in Supplementary Material S2B.

The plasmid construction steps were the same as those described above. Following the successful construction, lentiviral packaging was performed. After packaging was completed, the HT22 cells were infected. After cell passaging, the density reached 70%–80%, and drug screening continued. After the drug screening, the surviving cells were cultured in a purine medium.

### 2.14 Lentivirus packaging

The lentiviral packaging was performed when the HEK293FT cells reached 70–80% confluency. The lentiviral packaging complex was prepared as follows: Opti‐MEM, psPAX2, pMD2.G, and the plasmids were successively added, mixed, and incubated at room temperature for 5 min. PEI was then added, mixed, and incubated at room temperature for 20 min. The HEK293FT cells were digested and passaged at a 1:1 ratio. The above mixture was added to the cells, and the supernatant was collected after 48 h and filtered.

### 2.15 Co‐immunoprecipitation

Cell or tissue proteins were lysed using immunoprecipitation (IP) lysis buffer (containing 10% phosphatase inhibitor and 10% phenylmethyl sulfonyl fluoride). After mixing, the mixture was placed on ice for 30 min. The centrifugal supernatant was collected (4°C, 12000 *g*, 15 min) and placed on ice. Protein A/G magnetic beads (Beaver, China) were washed with IP binding buffer, then the corresponding IP antibody (50 μg/mL) was added and incubated for 30 min on a mixing rotator. Then, the previously prepared antigen samples were added, and the mixture was mixed with a pipette. After 1 h of reaction on the mixing rotator, magnetic separation was performed, and the mixture was washed with washing buffer. The immune complexes were boiled for 5 min with SDS sample buffer at 95°C and detected by western blotting.

### 2.16 Statistical analyses

The data were analyzed using GraphPad Prism 8.0. The Shapiro–Wilk test was performed on all data to confirm the normal distribution. Data that met normal distribution were analyzed using one‐way analysis of variance (ANOVA) and Tukey's test. Statistical significance was defined as *p* < 0.05. The mean ± SD was used to express values. Data that did not conform to normal distribution were analyzed using the rank sum test. *p* < 0.05 indicates a statistically significant difference in the mean rank between the two groups.

## 3 RESULTS

### 3.1 Time course and spatial expression of BRCC3 after MCAO and OGD/R

**FIGURE 1:**
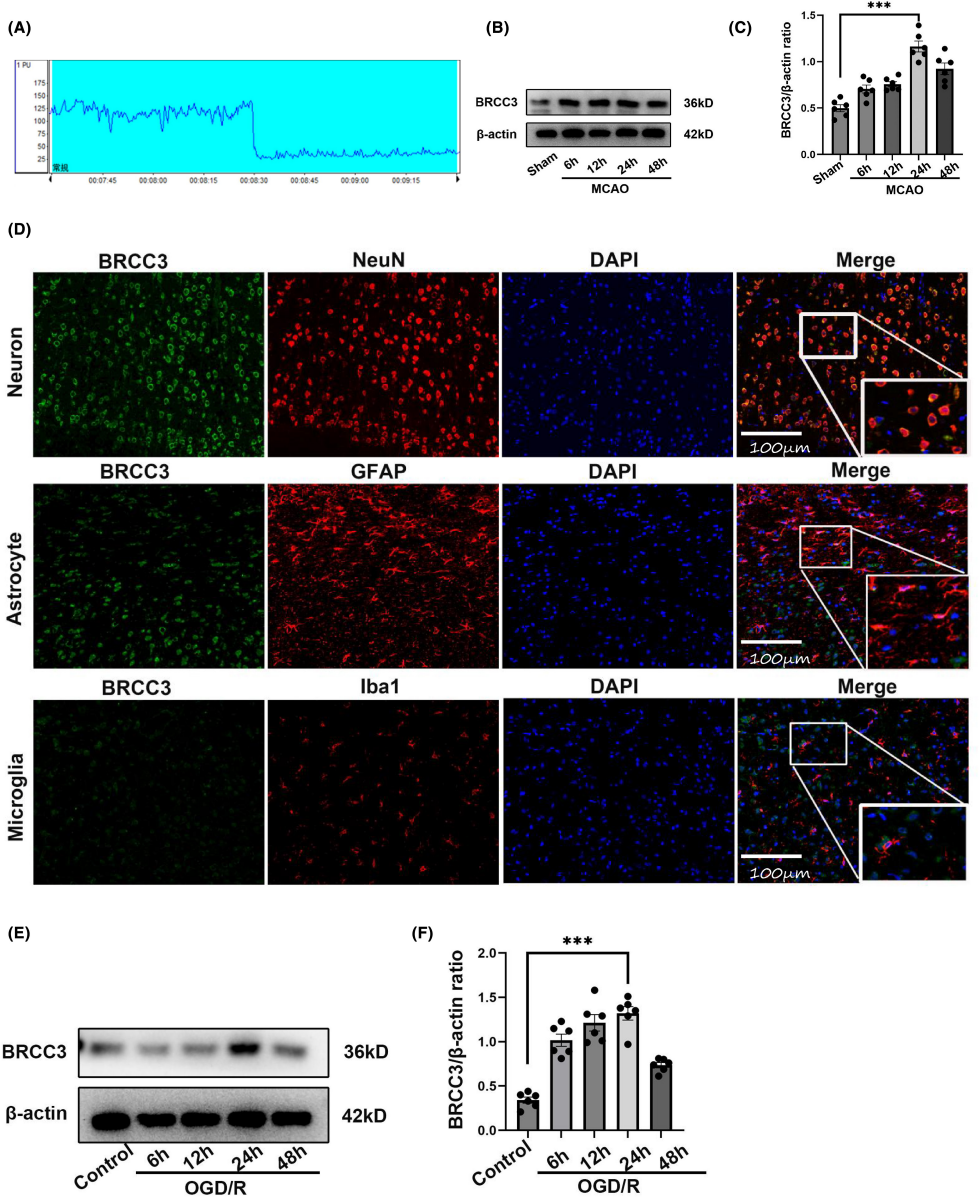
Time course and spatial expression of BRCC3 after MCAO and OGD/R. (A) Application of laser Doppler flowmeter in MCAO surgery for monitoring cerebral blood flow. (B, C) Western blotting analysis of BRCC3 in peri‐infarcted cortex of C57BL/6 mouse 6, 12, 24, and 48 h after MCAO reperfusion and Sham group (each group *n* = 6). (D) Immunofluorescence staining for detecting the localization of BRCC3 in neurons, astrocytes, and microglia (scale bar = 100 μm). (E) Protein level of BRCC3 in HT22 cells of OGD/R model was detected using western blot after 6, 12, 24, and 48 h of oxygen/glucose reperfusion compared with control group (each group *n* = 6). (F) Statistical diagram of the protein expression of BRCC3 in Figure E. Statistical data for each group are represented as mean ± SD. ***p < 0.001.

To determine whether BRCC3 is involved in brain I/R injury, we first monitored cerebral blood flow changes during the model process to ensure successful embolization (Figure 1A). Western blotting was then used to detect endogenous BRCC3 expression in the ipsilateral (left) cerebral cortex. As shown in Figure 1B,C, compared to the Sham group, the BRCC3 levels significantly increased at 6 h, peaked at 24 h after MCAO, and decreased at 48 h. As inflammation peaked after 24 h of reperfusion in the mouse brain ischemic disease process, we chose 24 h as the reperfusion time for subsequent studies. Immunofluorescence staining showed that BRCC3 was more abundant in the neurons than in microglia and astrocytes 24 h after MCAO (Figure 1D). As shown in Figure 1E,F, compared with those in the control group, the BRCC3 levels were significantly increased at 24 h after OGD/R and decreased at 48 h. Therefore, we also chose a 24 h reoxygenation time as the subsequent modeling time for the OGD/R model.

### 3.2 Impact of BRCC3 on neurological ability after the cerebral I/R injury

**FIGURE 2:**
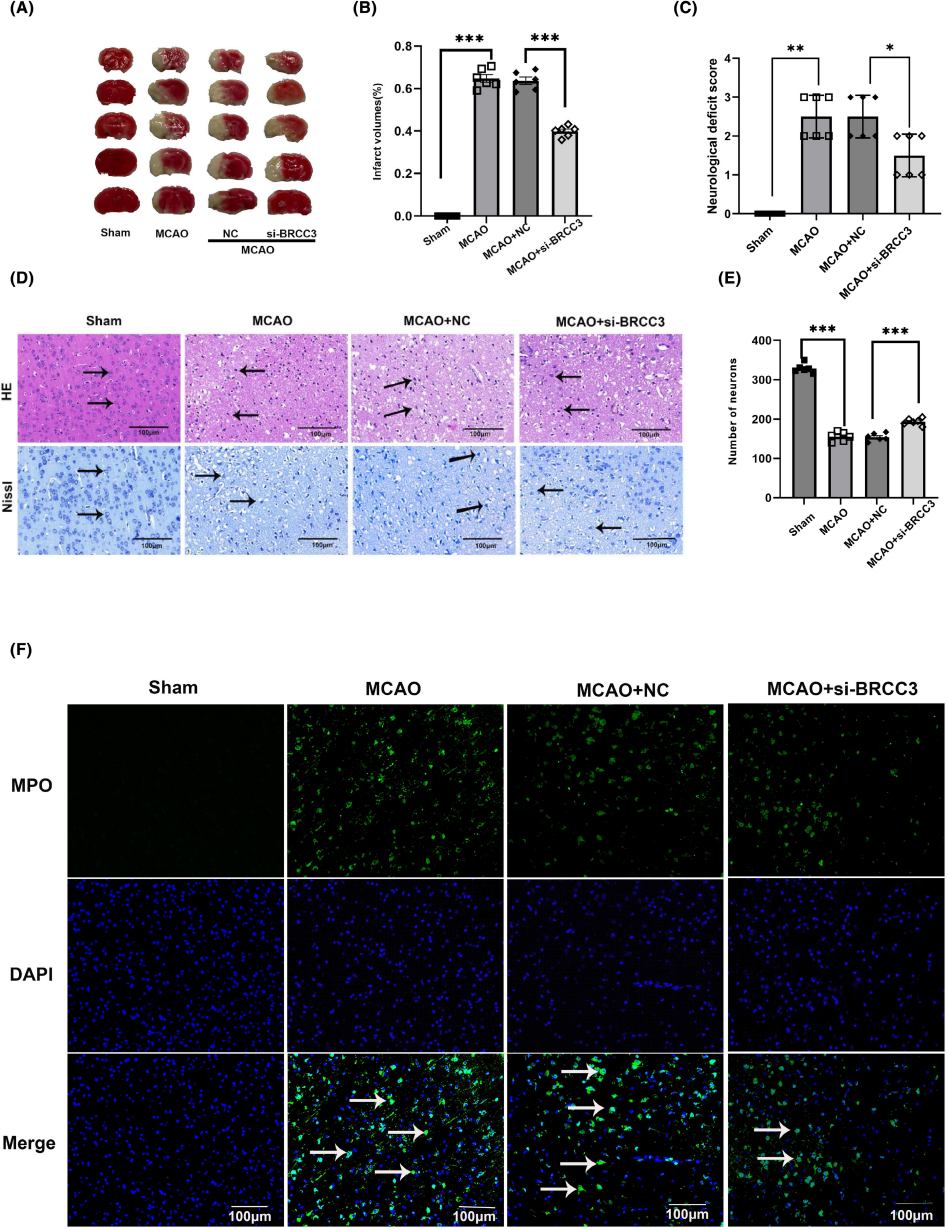
Impact of BRCC3 on neurological ability after cerebral I/R Injury. (A, B) Representative 2,3,5‐triphenyltetrazolium chloride (TTC) staining (*n* = 6). (C) Neurological score analysis (*n* = 6). (D–E) Representative HE and Nissl staining (scale bar = 100 μm) of peri‐infarcted cortex of MCAO with or without BRCC3 siRNA and Sham groups (*n* = 6). Arrows indicate representative neurons and Nissl bodies. (F) Representative MPO staining in Sham, MCAO, MCAO +NC, and MCAO+si‐BRCC3 groups (scale bar = 100 μm). Statistical data for each group are represented as mean ± SD. *p < 0.05, **p < 0.01, and ***p < 0.001.

To investigate the impact of BRCC3 siRNA after 24 h MCAO in mice, we tested the infarct area, neurological impairment scores, and HE and Nissl staining. Compared with the Sham group, the MCAO group showed significant neurological impairment. BRCC3 siRNA notably improved neurological impairment. In the TTC and neurological deficit score tests (Figure 2A–C), the MCAO group showed a remarkable increase in the infarct area and neurological impairment scores compared with those in the Sham group. However, a significant reduction was observed in the BRCC3 siRNA group. HE and Nissl staining (Figure 2D,E) showed that BRCC3 siRNA significantly improved edema and reversed the number of neurons. Myeloperoxidase (MPO) immunofluorescence staining was used to evaluate the inflammatory damage. As shown in Figure 2F, compared with that in the control group, the number of MPO‐positive cells around the cerebral cortex was drastically reduced after BRCC3 siRNA treatment. These results suggest that BRCC3 intervention can ameliorate nerve damage caused by cerebral I/R injury in mice.

### 3.3 Effect of BRCC3 on inflammation and pyroptosis after the cerebral I/R Injury

**FIGURE 3:**
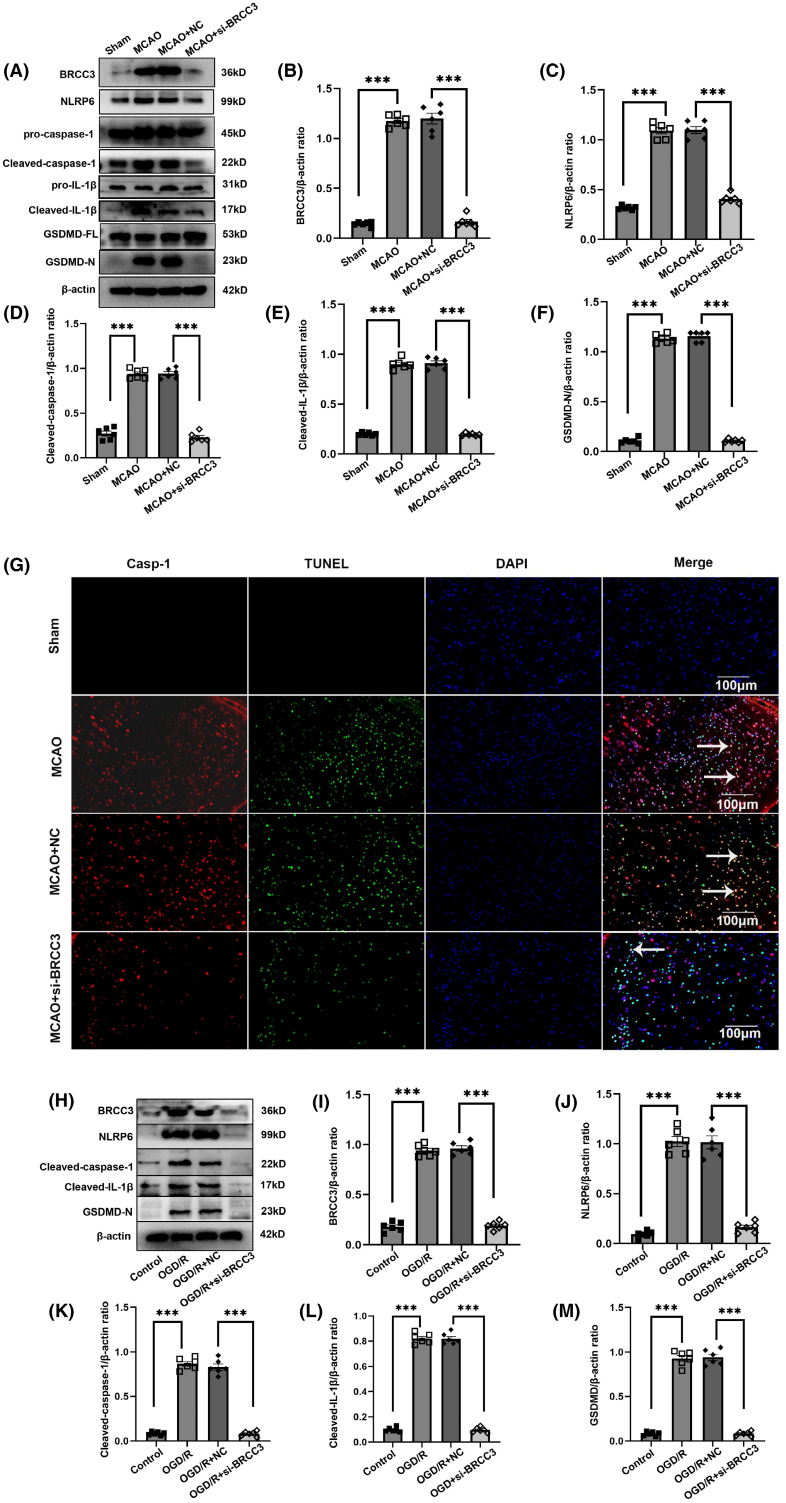
Effect of BRCC3 on inflammation and pyroptosis after cerebral I/R injury. (A–F) Western blotting analysis of BRCC3, NLRP6, cleaved‐caspase‐1, cleaved‐IL‐1β, and GSDMD‐N in peri‐infarcted cortex of C57BL/6 mouse in Sham, MCAO, MCAO+NC, and MCAO+si‐BRCC3 groups (*n* = 6). (G) Representative immunofluorescence staining (red: caspase‐1, Green: TUNEL, scale bar = 100 μm). Arrows indicate representative colocalization of positive cells (TUNEL and caspase‐1). (H–M) Western blotting analysis of BRCC3, NLRP6, cleaved‐caspase‐1, cleaved‐IL‐1β, and GSDMD‐N in HT22 cells in the Control, OGD/R, OGD/R + NC, and OGD/R + si‐BRCC3 groups (*n* = 6). Statistical data for each group are represented as mean ± SD. ***p < 0.001.

To determine if BRCC3 affects inflammation and pyroptosis during cerebral I/R injury, we monitored the expression of NLRP6, downstream inflammatory factors, and key components of pyroptosis after BRCC3 knockdown. Compared with that in the Sham group (Figure 3A–F), the MCAO group had increased expression of NLRP6, downstream inflammatory cytokines, and pyroptosis, and BRCC3 siRNA notably decreased the expression of NLRP6, cleaved‐caspase‐1, cleaved‐IL‐1β, and GSDMD‐N. These results indicated that BRCC3 intervention can effectively reduce inflammation and pyroptosis after cerebral I/R injury. Immunofluorescence staining for caspase‐1 and terminal deoxynucleotidyl transferase (TdT) dUTP nick‐end labeling (TUNEL) assay revealed that the amount of TUNEL‐ and caspase‐1‐positive cells (pyroptosis) remarkably increased in the MCAO group compared to that in the Sham group (Figure 3G). Compared to in the MCAO+NC group, pyroptosis in the BRCC3 siRNA group significantly reduced. Simultaneously, we employed OGD/R to simulate the cerebral I/R injury model in vitro and detected the relevant inflammatory and pyroptosis indicators (Figure 3H–M), consistent with those in vivo. In conclusion, endogenous BRCC3 influences inflammation and pyroptosis following cerebral I/R injury.

### 3.4 Influence of BRCC3 on downstream inflammation and pyroptosis through NLRP6 inflammasome

**FIGURE 4:**
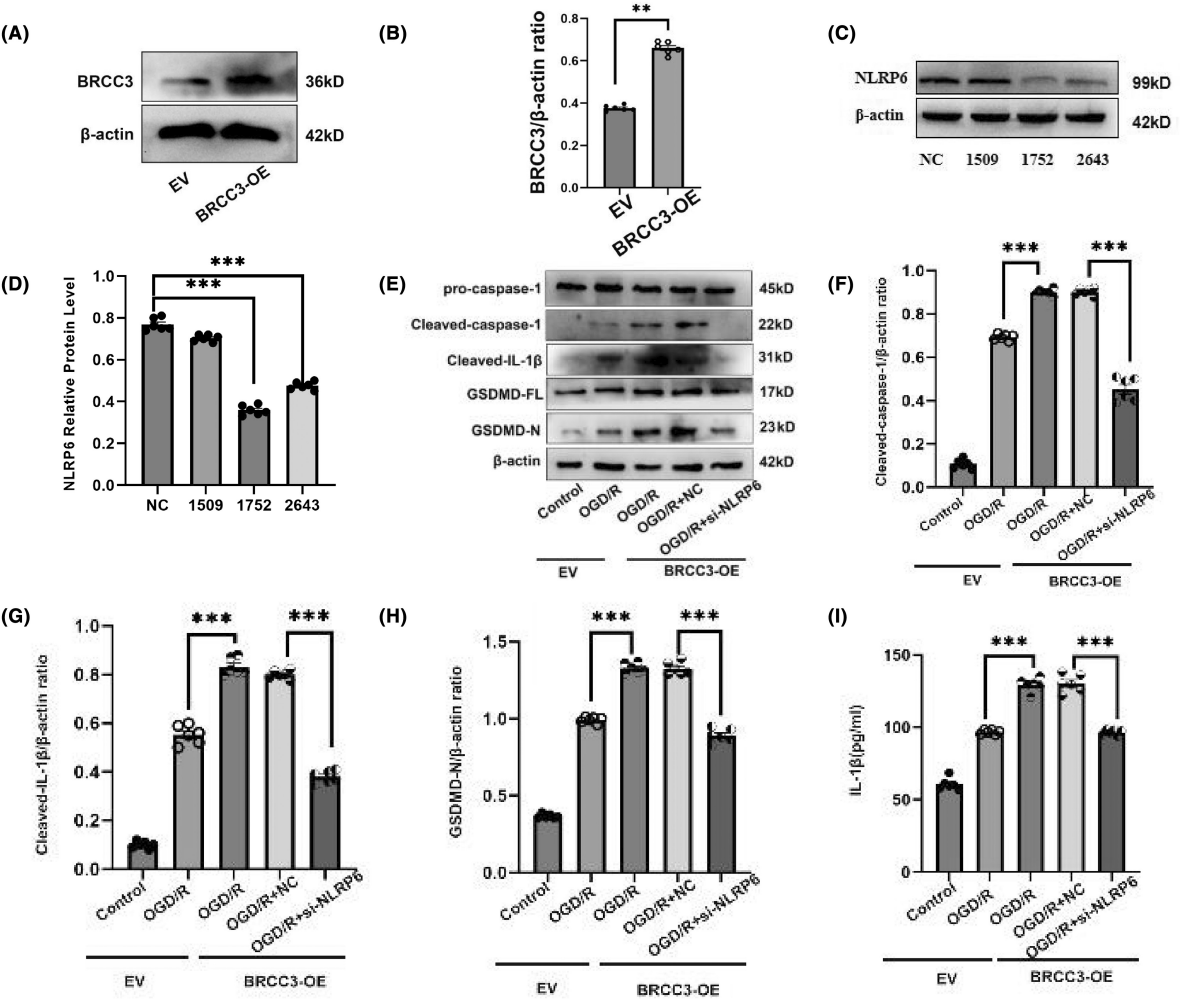
Influence of BRCC3 on downstream inflammation and pyroptosis through NLRP6 inflammasome. (A and B) Detection of BRCC3 protein levels via western blotting after overexpression of BRCC3 in HT22 cells infected with lentivirus (*n* = 6).(C and D) Most efficient NLRP6 siRNA from three interference fragments in vivo was screened by western blotting (*n* = 6). (E–H) Protein expression of cleaved‐caspase‐1, cleaved‐IL‐1β, and GSDMD‐N was tested via western blotting after the overexpression of BRCC3 and treatment with NLRP6 siRNA in HT22 cells (*n* = 6). (I) ELISA was performed to measure secretion of IL‐1β after the overexpression of BRCC3 and treatment with NLRP6 siRNA in HT22 cells (*n* = 6). Statistical data for each group are represented as mean ± SD. **p < 0.01; ***p < 0.001.

To assess whether BRCC3 regulates downstream inflammation and pyroptosis through the NLRP6 inflammasome, we obtained stably overexpressing cells, transfected NLRP6 siRNA, and detected changes in downstream‐related factors after infecting HT22 cells with a lentiviral plasmid overexpressing BRCC3. First, we identified the overexpression efficiency of BRCC3 (Figure 4A,B), further screened the knockdown fragment of NLRP6, and selected the 1752 fragment with the highest knockdown efficiency for subsequent experiments (Figure 4C,D). The OGD/R + EV group had higher cleaved‐caspase‐1, cleaved‐IL‐1β, and GSDMD‐N expression than the control group (Figure 4E–H). However, after the overexpression of BRCC3, compared with that in the OGD/R + EV group, the expression of cleaved‐caspase‐1, cleaved‐IL‐1β, and GSDMD‐N in the OGD/R + BRCC3‐OE group increased. Interestingly, OGD/R + si‐NLRP6 + BRCC3‐OE treatment reversed the increase in expression of cleaved‐caspase‐1, cleaved‐IL‐1β, and GSDMD‐N in the OGD/R + BRCC3‐OE group. The enzyme‐linked immunosorbent assay results of Il‐1β were consistent with the above (Figure 4I). Therefore, NLRP6 downregulation reverses the effects of BRCC3 overexpression on inflammation and pyroptosis. In conclusion, BRCC3 regulates inflammation and pyroptosis during cerebral I/R injury through NLRP6.

### 3.5 BRCC3 interacts with NLRP6

**FIGURE 5:**
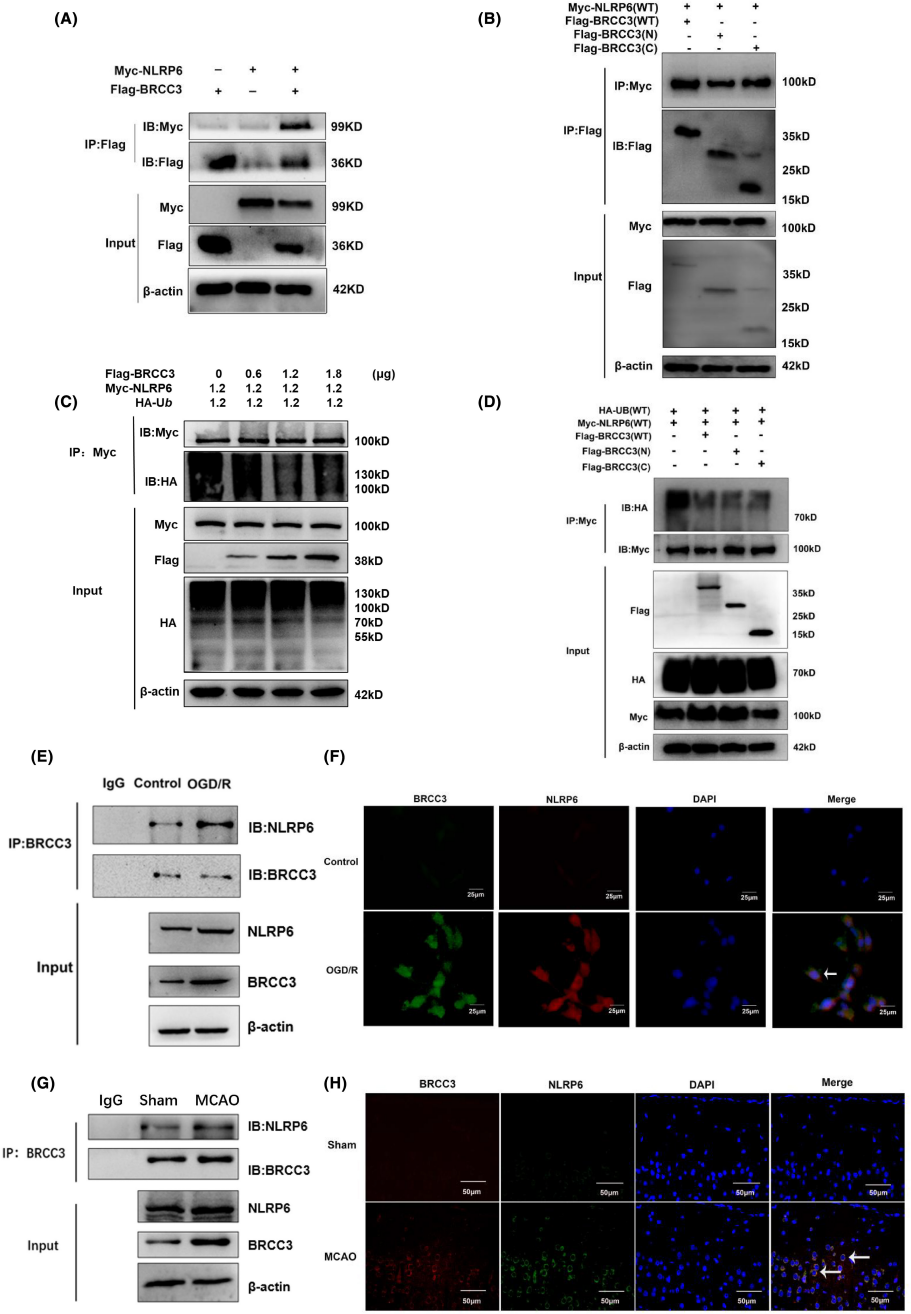
BRCC3 interacts with NLRP6. (A) CO‐IP analysis of interaction between BRCC3 and NLRP6 in HEK293T cells by transfecting NLRP6 and BRCC3 plasmids. (B) CO‐IP analysis of interaction between full‐length or truncated BRCC3 and NLRP6 in HEK293T cells by transfecting NLRP6 and full‐length or truncated BRCC3 plasmids. (C) IP analysis of NLRP6 ubiquitination by transfecting BRCC3, NLRP6, and ubiquitin plasmids in HEK293T cells. (D) IP analysis of NLRP6 ubiquitination by transfecting NLRP6, full‐length or truncated BRCC3, and ubiquitin plasmids in HEK293T cells. (E) CO‐IP analysis of interaction between BRCC3 and NLRP6 in HT22 cells of the Control and OGD/R groups.(F) Immunofluorescence analysis of BRCC3 and NLRP6 localization in HT22 cells of the Control and OGD/R groups. (G) CO‐IP analysis of interaction between BRCC3 and NLRP6 in the Sham and MCAO group. (H) Immunofluorescence analysis of BRCC3 and NLRP6 localization in the Sham and MCAO groups.

BRCC3 reportedly monitors downstream inflammation and pyroptosis through the NLRP6 inflammasome. We investigated the relationship between BRCC3 and NLRP6 expression. We transfected BRCC3 and NLRP6 plasmids into HEK293T cells and examined their interactions. The results showed an interaction between BRCC3 and NLRP6, and the N‐terminal and C‐terminal of BRCC3 were involved (Figure 5A,B). As BRCC3 regulates ubiquitination, we tested whether BRCC3 regulated the ubiquitination level of NLRP6. We found that BRCC3 decreased the ubiquitination of NLRP6 in HEK293T cells and that this effect was associated with both the N‐ and C‐terminal of BRCC3 (Figure 5C,D). Subsequently, we detected the interaction between BRCC3 and NLRP6 in vitro and in vivo (Figure 5E–H). The results showed that BRCC3 and NLRP6 colocalized. We further examined whether they interacted with CO‐IP; BRCC3 interacted with NLRP6, and the interaction increased significantly in the I/R model, both in vitro and in vivo.

### 3.6 Effect of BRCC3 on the activation of NLRP6 inflammasome

**FIGURE 6:**
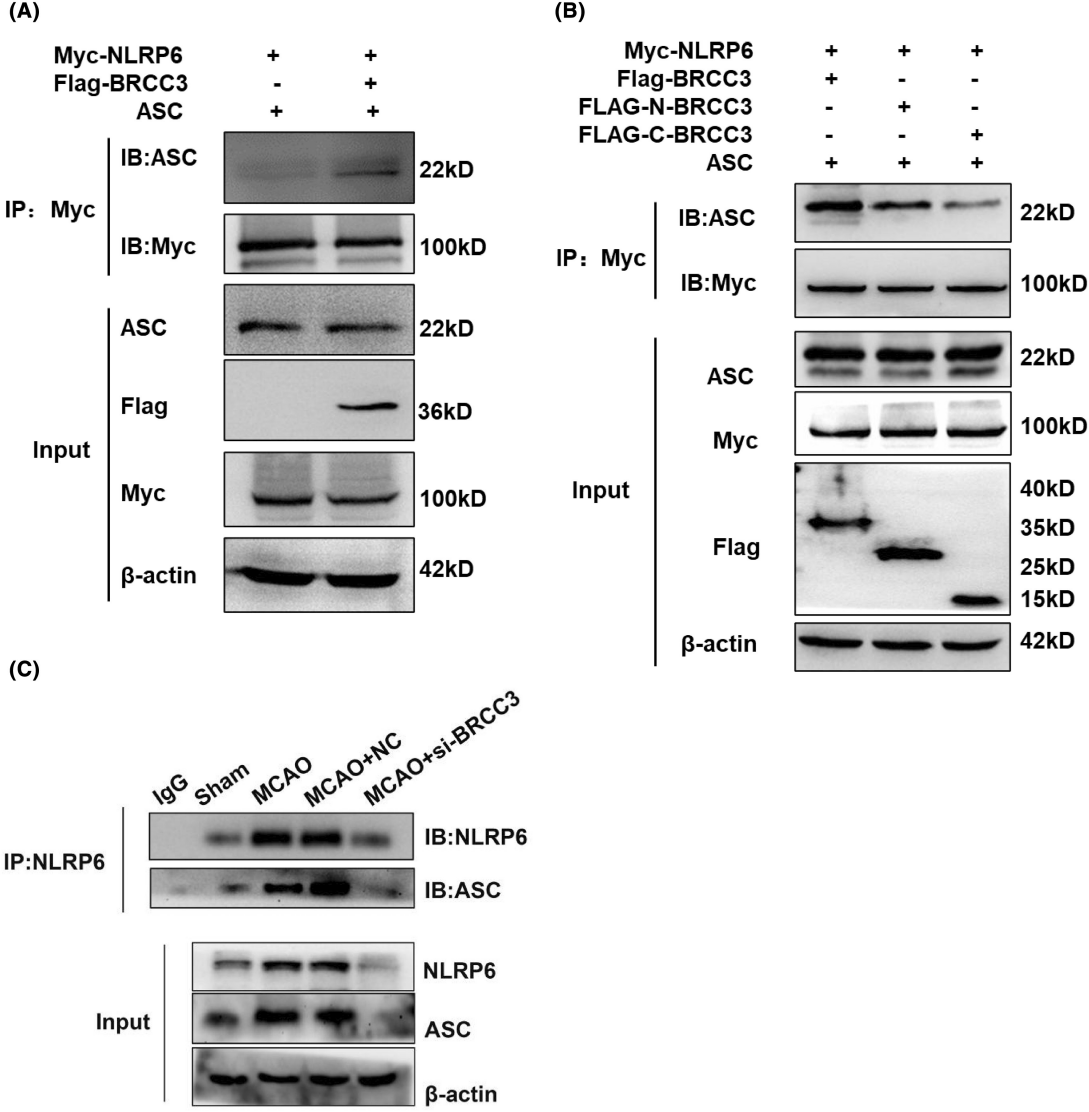
Effect of BRCC3 on the activation of NLRP6 inflammasome. (A) CO‐IP analysis of binding between ASC and NLRP6 in HEK293T cells by transfecting NLRP6, BRC3, and ASC plasmids. (B) CO‐IP analysis of binding between ASC and NLRP6 in HEK293T cells by transfecting NLRP6, full‐length or truncated BRCC3, and ASC plasmids. (C) CO‐IP analysis of binding between ASC and NLRP6 in the Sham, MCAO, MCAO+NC, and MCAO+si‐BRCC3 groups.

Finally, as the assembly of NLRP6 inflammatory bodies reflects NLRP6 inflammasome activation, we further explored the effect of BRCC3 in activating the NLRP6 inflammasome. The presence of BRCC3 significantly improved the interaction between NLRP6 and ASC in HEK293T cells by comparing its N‐terminal and C‐terminal (Figure 6A,B). Compared with the Sham group, MCAO notably enhanced the binding of NLRP6 and ASC, whereas the BRCC3 siRNA significantly decreased the interaction between NLRP6 and ASC (Figure 6C).

## 4 DISCUSSION

I/R induces early‐stage brain damage associated with oxidative stress, calcium overload, oxygen‐free radical damage, and inflammatory cascade response.^26^, ^27^ Here, the BRCC3 knockdown improved neurological deficits and inflammation, which could lead to reduced brain damage.

This study is the first attempt to investigate the effect of BRCC3 on nerve injury and its potential mechanisms in cerebral I/R in mice. Our results revealed that: (1) BRCC3 expression was upregulated 24 h after MCAO and OGD/R, and BRCC3 in mouse brain tissue is mainly distributed in neurons; (2) BRCC3 knockdown improved neurological function 24 h after MCAO; (3) BRCC3 knockdown improved inflammation and pyroptosis both in vivo and in vitro; (4) in vitro, NLRP6 knockdown reversed the impact of BRCC3 overexpression on inflammation and pyroptosis; (5) BRCC3 interacts with NLRP6 in vivo and in vitro, both at the N‐ and C‐terminal. Simultaneously, BRCC3 affected the ubiquitination of NLRP6, and (6) BRCC3 promoted NLRP6 inflammasome assembly.

Cerebral I/R injury induces brain edema, neurological deficits, and neuroinflammation, the primary pathological changes and factors contributing to poor prognosis after MCAO. BRCC3 is extensively expressed in the brain, heart, muscles, kidneys, and small intestine.^28^ BRCC3 is involved in several brain diseases and plays a significant role.^19^, ^20^, ^29^ Here, we observed an increase in endogenous BRCC3 expression, soon after MCAO and OGD/R, and BRCC3 was mainly expressed in neurons. This result was consistent with previous findings in a mouse model of Parkinson's disease.^19^

The NLRP6 inflammasome regulates inflammation during cerebral I/R injury and intracerebral hemorrhage (ICH).^30^ IL‐1β, IL‐18, and GSDMD all act downstream of the NLRP6 inflammasome.^31^, ^32^ BRCC3 reportedly influences NLRP6 inflammasome activation through interaction with NLRP6 in rats.^20^ The results of this study indicate that endogenous BRCC3 siRNA significantly downregulated the protein expression of NLRP6, cleaved‐caspase‐1, cleaved‐IL‐1β, and GSDMD‐N and reduced neutrophil infiltration, thus improving neurological deficit after MCAO in mice. Conversely, BRCC3 overexpression increased the protein expression of cleaved‐caspase‐1, cleaved‐IL‐1β, and GSDMD‐N; NLRP6 siRNA reversed these effects of BRCC3‐OE. Our findings indicated that the effect of BRCC3 on neuroinflammation and pyroptosis is associated with NLRP6 inflammasome activation in cerebral I/R injury.

The specific regulatory relationship between BRCC3 and NLRP6 inflammasome is yet to be determined. The deubiquitination enzyme Cyld can prevent excessive IL‐18 production in the colon mucosa via deubiquitination of NLRP6 and inhibition of NLRP6‐ASC binding, which regulates IL‐18 maturation.^33^ Notably, BRCC3 deubiquitinated NLRP6; however, BRCC3 facilitated NLRP6 and ASC assembly, inconsistent with the role of Cyld. Moreover, the knockdown or overexpression of BRCC3 significantly affected the expression of NLRP6, whereas Cyld showed no effect on the NLRP6 expression. Therefore, the effect of BRCC3 on NLRP6 may be inconsistent with that of Cyld, requiring further study. Previous literature has shown that BRCC3 has deubiquitination function,^34^ but only the full‐length function of BRCC3 has been studied, but the specific domain of BRCC3 has not been studied. Therefore, according to the structural characteristics of BRCC3, we constructed N‐terminal and C‐terminal truncated bodies to explore the N‐terminal and C‐terminal deubiquitination function. The results show that both N‐terminal and C‐terminal can play a deubiquitination role. The MPN domain located at the N‐terminal is a catalytic domain with deubiquitinase activity,^35^ which can directly affect the ubiquitination level of NLRP6. However, there is currently no research indicating the specific function and role of C‐terminal, which may affect the ubiquitination of NLRP6 through direct or indirect catalysis.^36^ Later, we will further explore the reasons for the deubiquitination function of both the N‐terminal and C‐terminal.

Our study had some limitations. BRCC3 is implicated in several pathological processes, such as DNA damage repair, cell apoptosis, angiogenesis, tumorigenesis, bone marrow hyperplasia, myocardial injury, and inflammation.^29^, ^34^ Here, we examined the effects of brain damage caused by BRCC3 on neuroinflammation and pyroptosis after MCAO in mice. Therefore, we cannot rule out the possibility that BRCC3 mediates other damaging effects in cerebral I/R. Whether BRCC3 affects NLRP6 through deubiquitination remains unclear and requires further study.

Our findings indicate that BRCC3 may be a novel target for ameliorating cerebral I/R injury and alleviating the inflammatory response and pyroptosis induced by the NLRP6 inflammasome. The potential mechanism by which BRCC3 activates the NLRP6 inflammasome has been preliminarily discussed.

## AUTHOR CONTRIBUTIONS

XH: literature search, experimental design, investigation, and manuscript drafting. JT: investigation, analysis, data acquisition, and manuscript drafting. YJ: investigation, manuscript revision, and statistical analysis. JL: manuscript revision. YZ and JZ: technical and material support and experimental supervision.

## FUNDING INFORMATION

This work was supported by the National Natural Science Foundation of China (No. 81771261 and 82071305).

## CONFLICT OF INTEREST STATEMENT

The authors declare that they have no conflicts of interest.

## Supporting information


Data S1–S2

## Data Availability

The data that support the findings of this study are openly available in PUBMED at https://pubmed.ncbi.nlm.nih.gov/.
