# Supplementary material for: BRCC3 mediates inflammation and pyroptosis in cerebral ischemia/reperfusion injury by activating the NLRP6 inflammasome
Source: CNS Neurosci Ther. 2024 Mar 28;30(3):e14697. doi: 10.1111/cns.14697 (PMC10973773; doi:10.1111/cns.14697)
Supplement: Supplementary file 1 — Data S1–S2 [file CNS-30-e14697-s001.zip › Flow chart.docx]

Flow chart. Experimental design and animal groups. BRCC3, BRCA1-BRCA2-containing complex subunit 3; MCAO, middle cerebral artery occlusion/reperfusion; NC, negative Control; HE/Nissl, hematoxylin–eosin staining/Nissl staining; TTC, 2,3,5-triphenyltetrazolium chloride staining; IF, immunofluorescence; WB, western blot; OGD, oxygen-glucose deprivation; and OE, overexpression.
