# Supplementary material for: BRCC3 mediates inflammation and pyroptosis in cerebral ischemia/reperfusion injury by activating the NLRP6 inflammasome
Source: CNS Neurosci Ther. 2024 Mar 28;30(3):e14697. doi: 10.1111/cns.14697 (PMC10973773; doi:10.1111/cns.14697)
Supplement: Supplementary file 1 — Data S1–S2 [file CNS-30-e14697-s001.zip › original gels.pdf]

Figure 1B

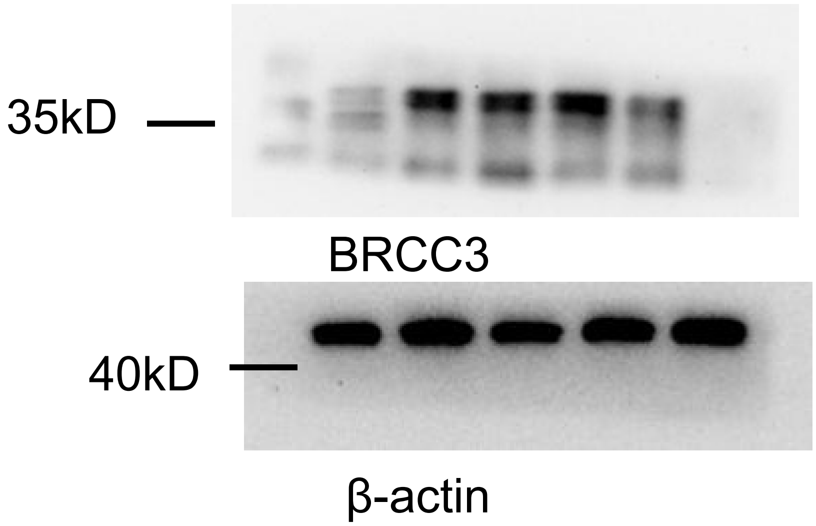

Figure 1E

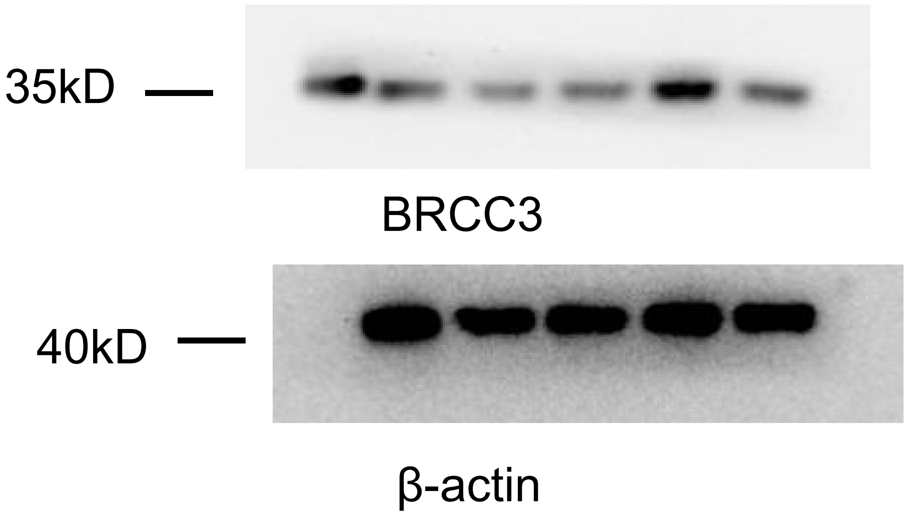

Figure 3A

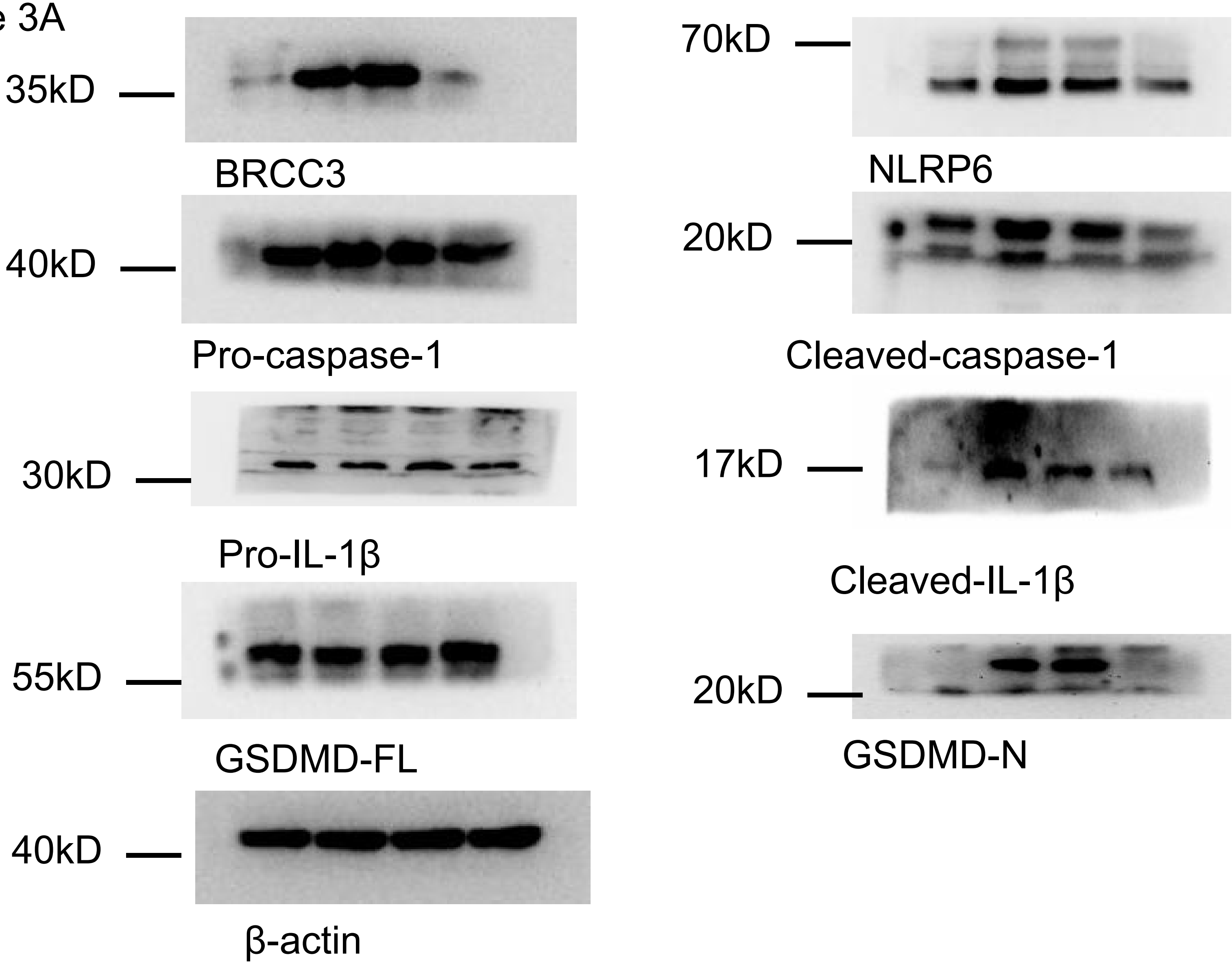
35kD
BRCC3
40kD
Pro-caspase-1
30kD
Pro-IL-1β
55kD
GSDMD-FL
40kD
β-actin
70kD
NLRP6
20kD
Cleaved-caspase-1
17kD
Cleaved-IL-1β
20kD
GSDMD-N

Figure 3H

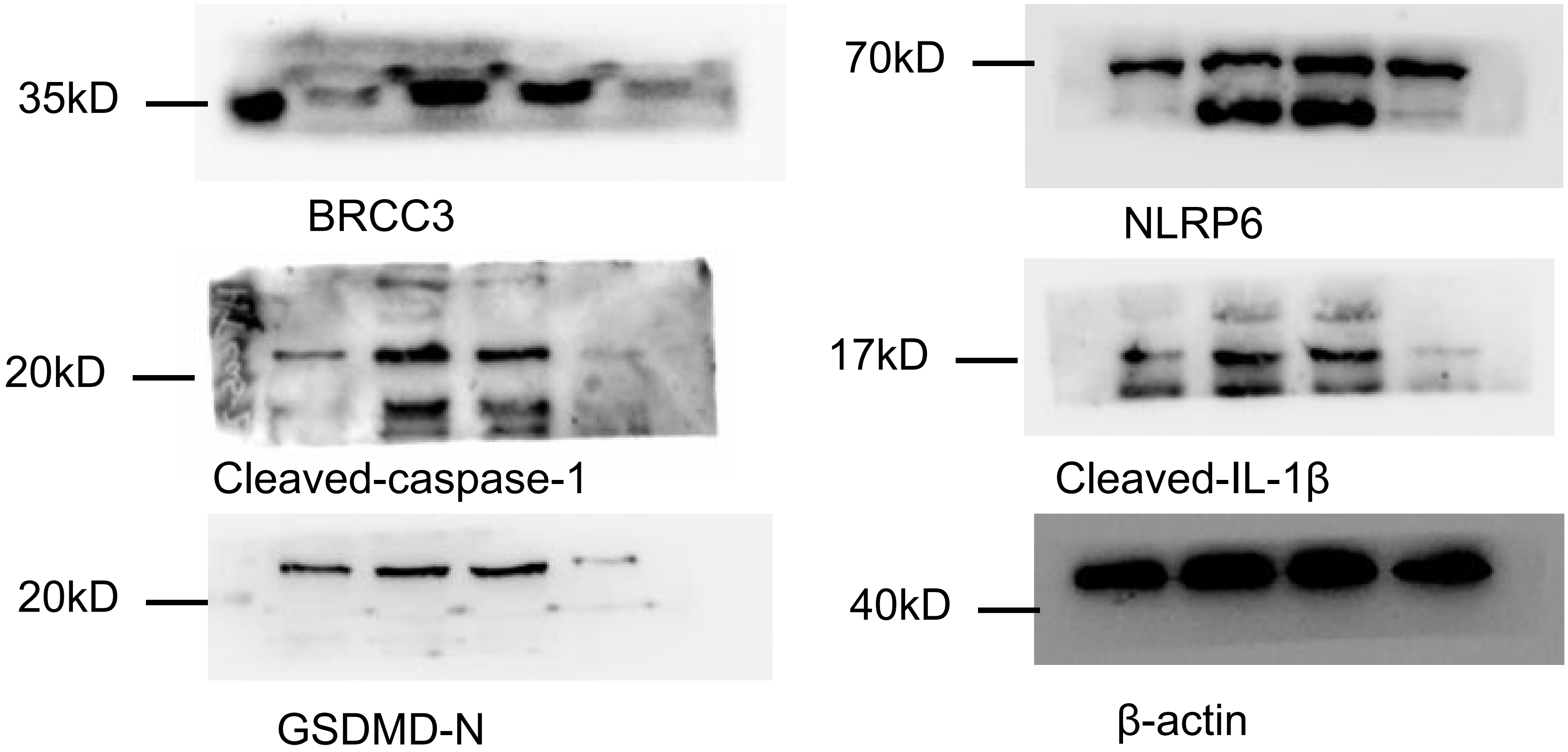
35kD
BRCC3
70kD
NLRP6
20kD
Cleaved-caspase-1
17kD
Cleaved-IL-1β
20kD
GSDMD-N
40kD
β-actin

Figure 4A

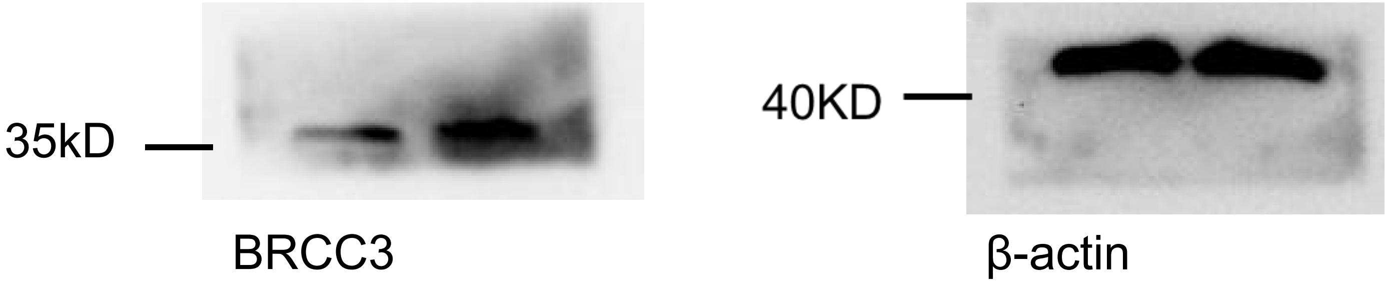

Figure 4C

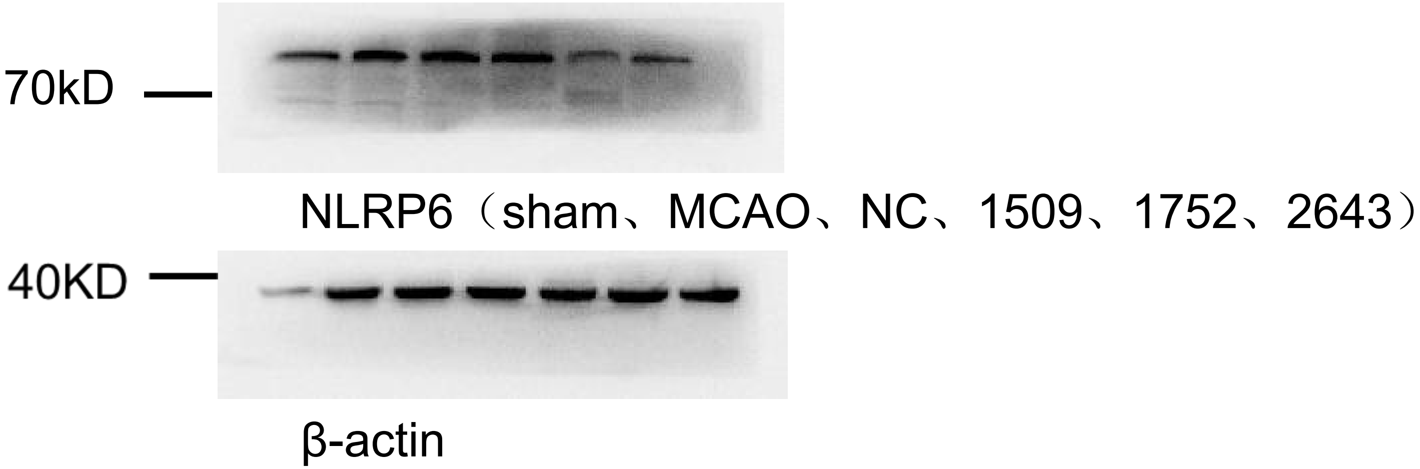

Figure 4E

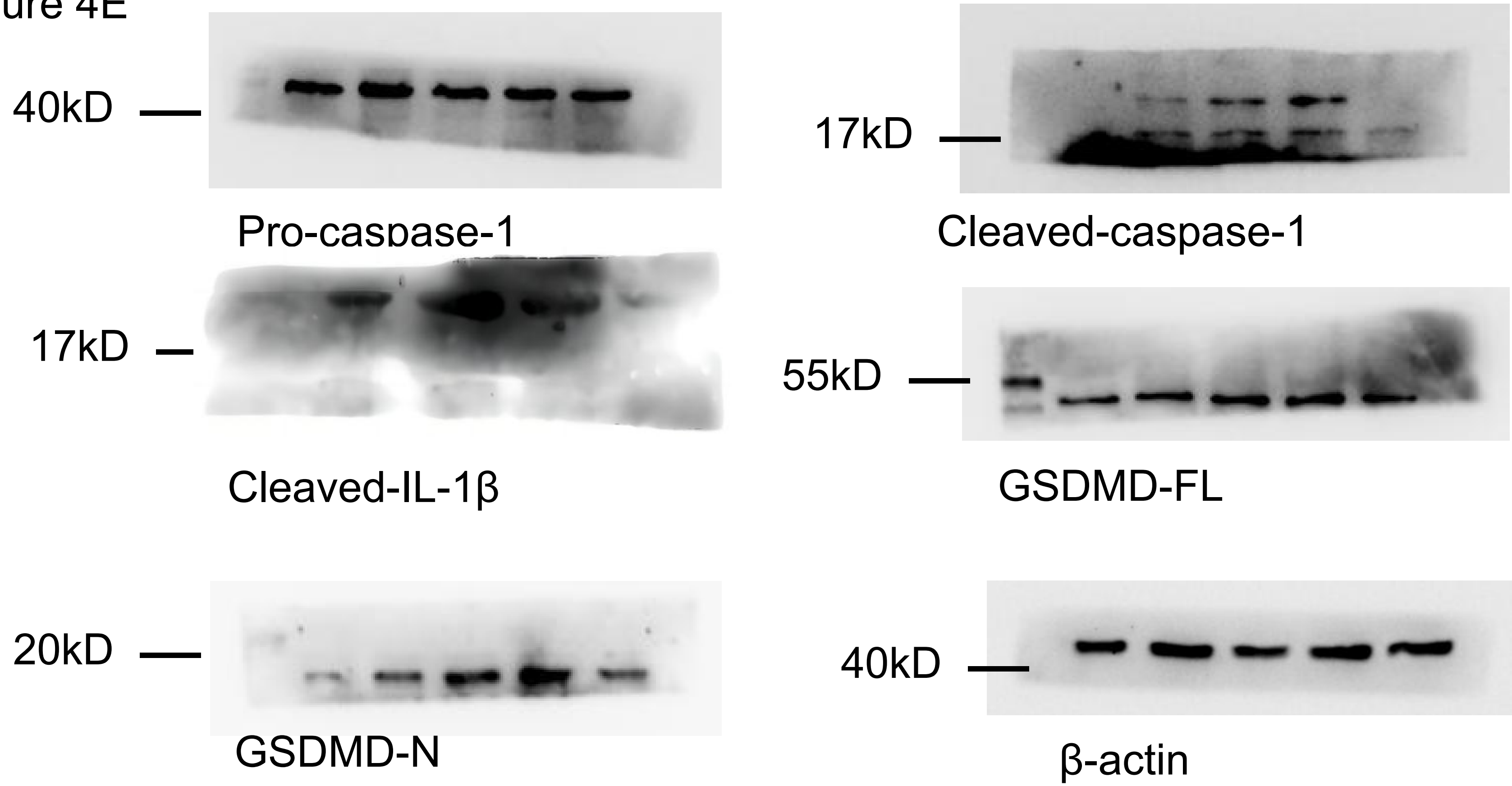

40kD
Pro-caspase-1
17kD
Cleaved-caspase-1
17kD
Cleaved-IL-1β
55kD
GSDMD-FL
20kD
GSDMD-N
40kD
β-actin

Figure 5A

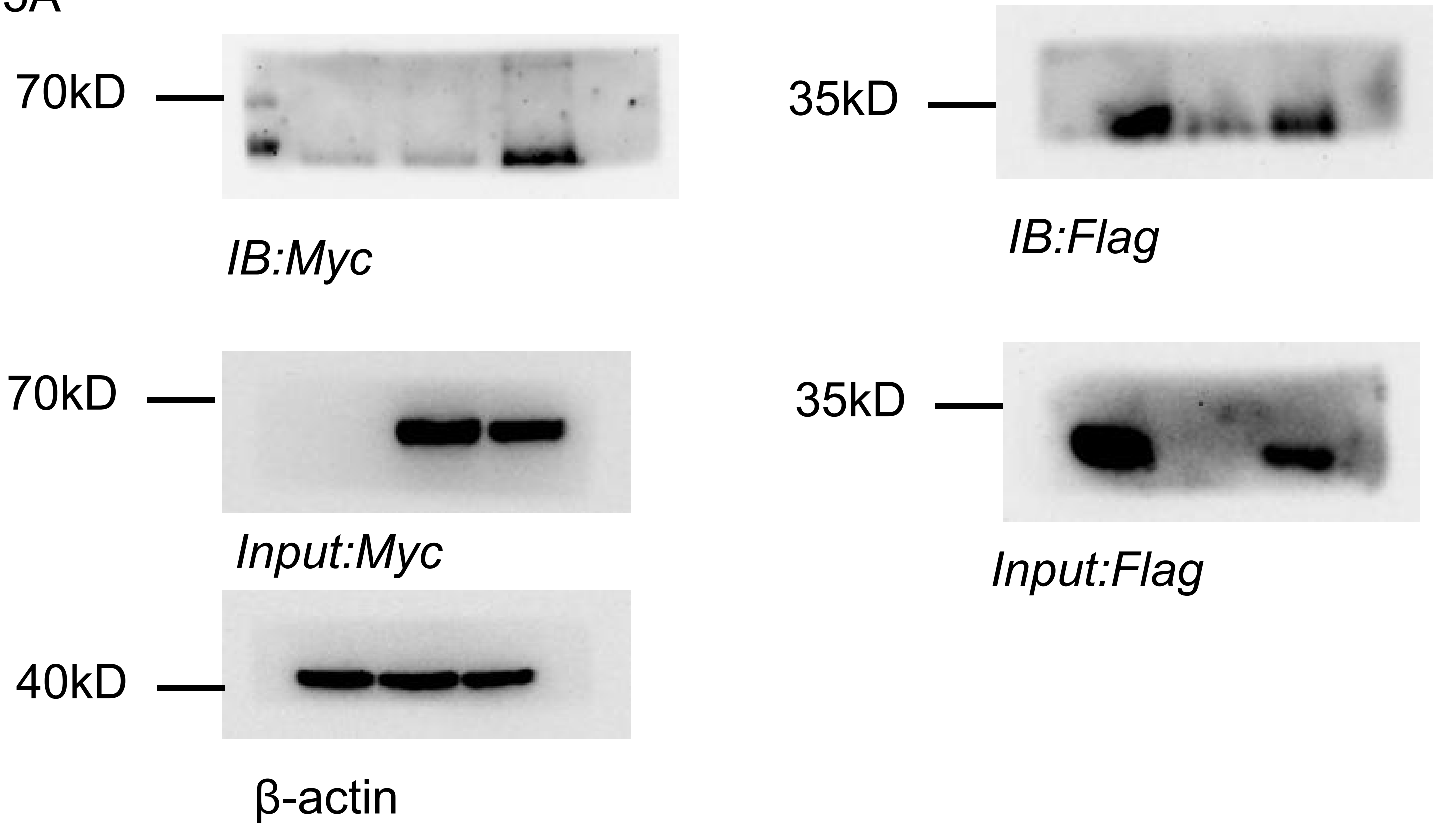
70kD
IB:Myc
35kD
IB:Flag
70kD
Input:Myc
35kD
Input:Flag
40kD
β-actin

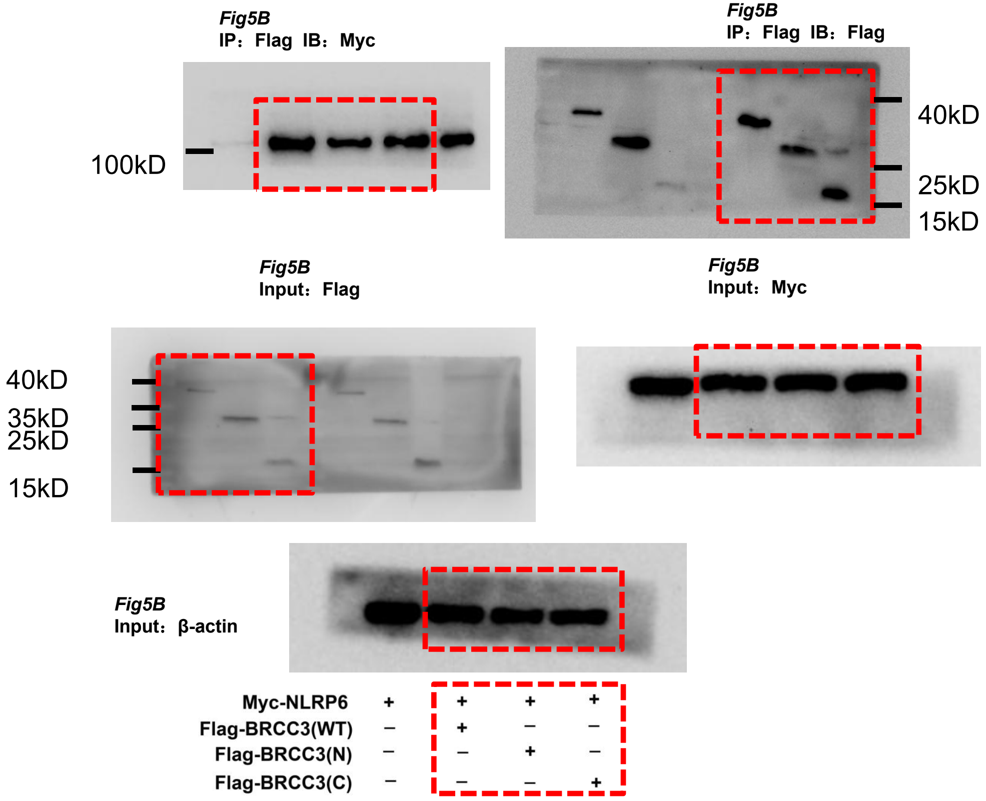
Fig5B
IP：Flag IB：Myc
100kD
Fig5B
IP：Flag IB：Flag
40kD
25kD
15kD
Fig5B
Input：Flag
40kD
35kD
25kD
15kD
Fig5B
Input：Myc
Fig5B
Input：β-actin
Myc-NLRP6 + + + +
Flag-BRCC3(WT) – + – –
Flag-BRCC3(N) – – + –
Flag-BRCC3(C) – – – +

Fig5C
IP：Myc IB：HA

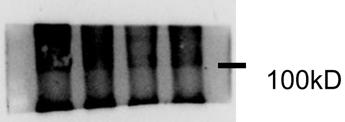

Fig5C
IP：Myc IB：Myc

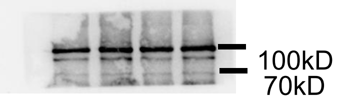

Fig5C
Input：Myc

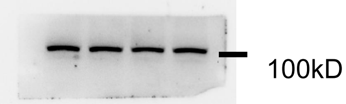

Fig5C
Input：Flag

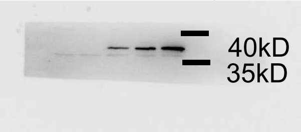

Fig5C
Input：β-actin

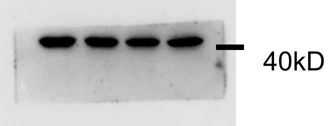

Fig5C
Input：HA

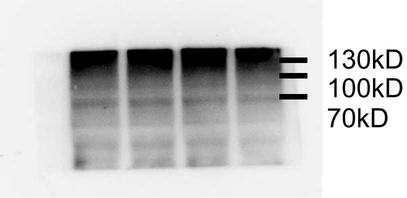

Figure 5D

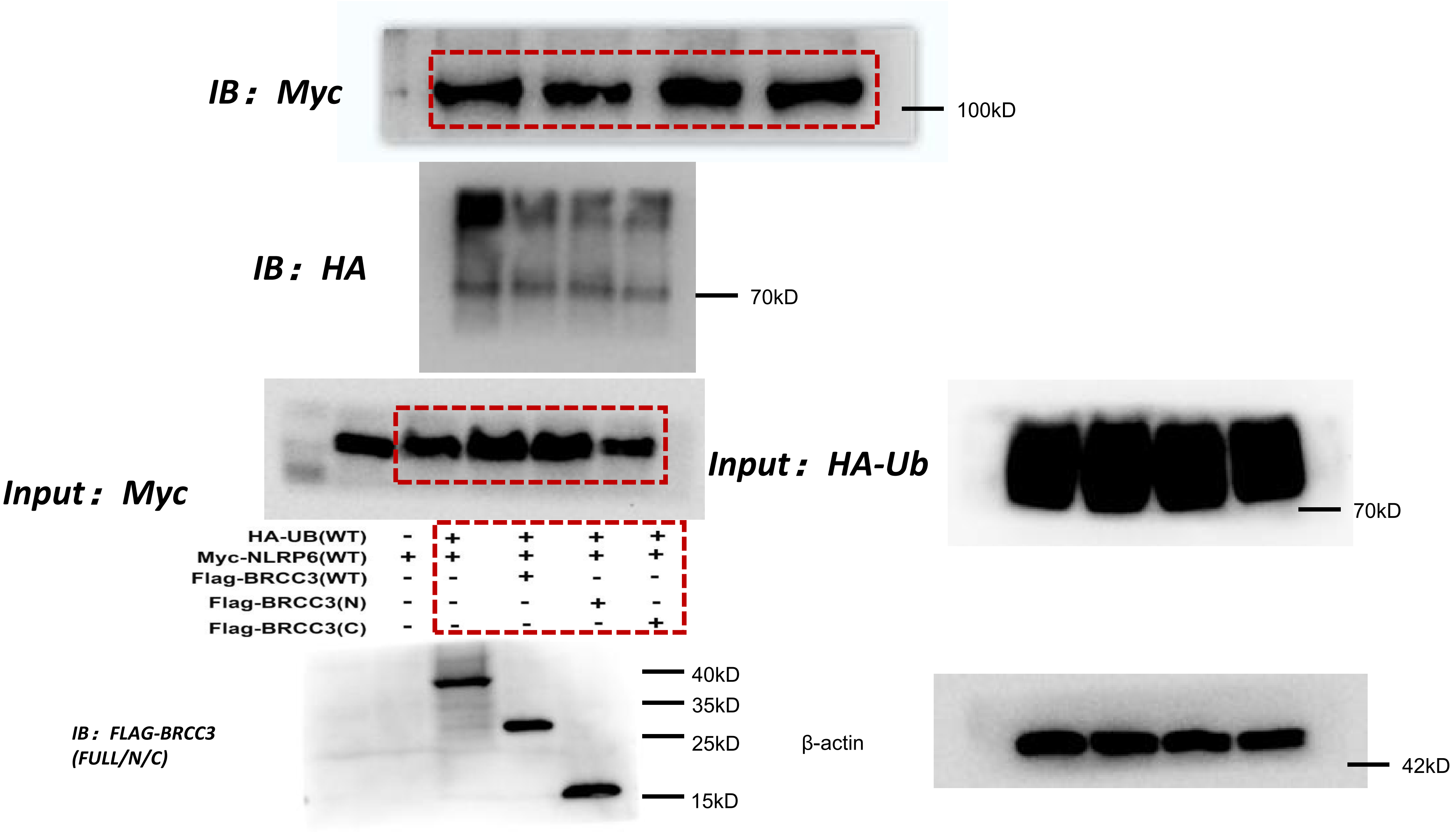

IB : Myc
100kD
IB : HA
70kD
Input : Myc
Input : HA-Ub
70kD
HA-UB(WT) - + + + +
Myc-NLRP6(WT) + + + + +
Flag-BRCC3(WT) - - + - -
Flag-BRCC3(N) - - - + -
Flag-BRCC3(C) - - - - +
IB : FLAG-BRCC3 (FULL/N/C)
40kD
35kD
25kD
15kD
β-actin
42kD

Fig5E
IP:BRCC3 IB:NLRP6

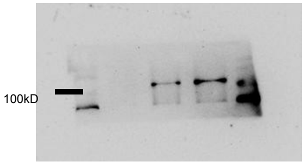

Fig5E
IP:BRCC3 IB:BRCC3

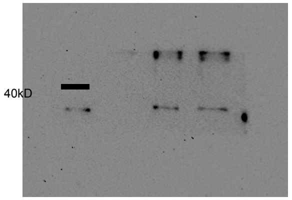

Fig5E
Input：NLRP6

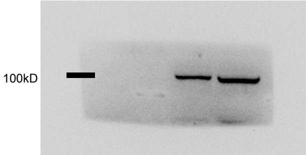

Fig5E
Input：BRCC3

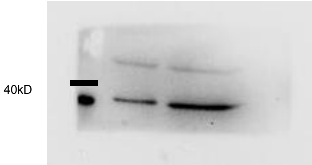

Fig5E
Input：*β-actin*

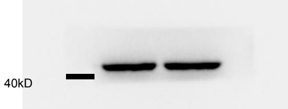

Fig5G
Input：*NLRP6*

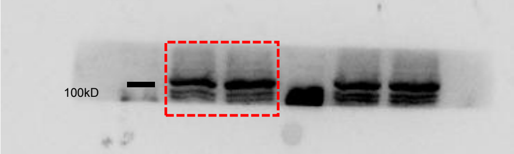

Fig5G
IP:BRCC3 IB:NLRP6

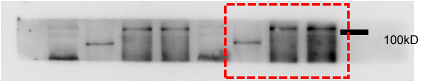

Fig5G
Input：*β-actin*

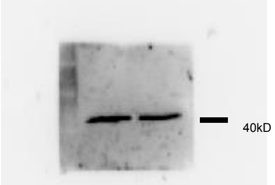

Fig5G
IP:BRCC3 IB:BRCC3

Fig5G
Input：*BRCC3*

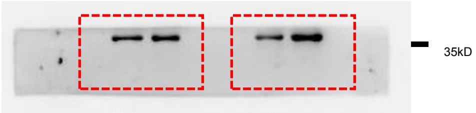

Fig6A
IP:Myc IB:Myc

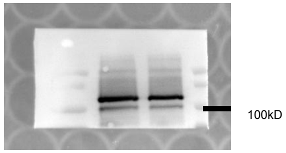

Fig6A
IP:Myc IB:ASC

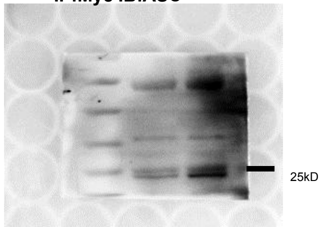

Fig6A
Input：*Flag*

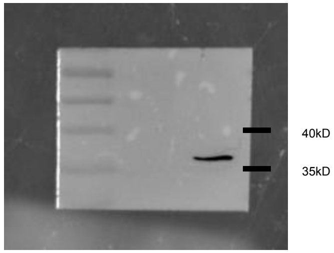

Fig6A
Input：*ASC*

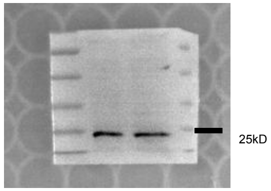

Fig6A
Input ： *Myc*

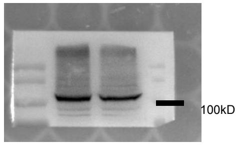

Fig6A
Input ： *β-actin*

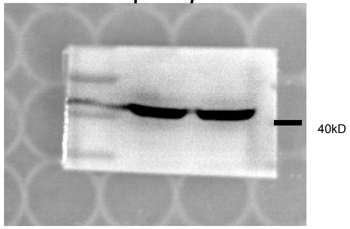

Fig6B
IP:Myc IB:ASC

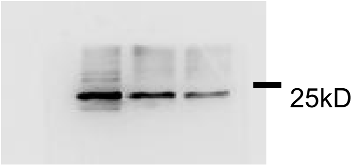

Fig6B
IP:Myc IB:Myc

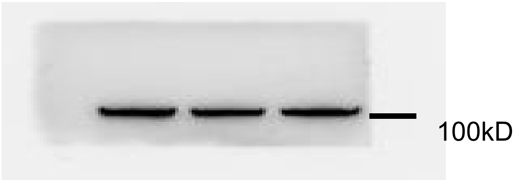

Fig6B
Input：*Flag*

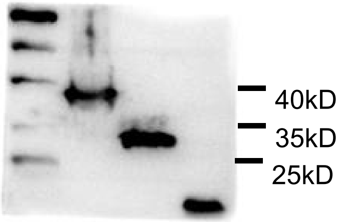

Fig6B
Input：*ASC*

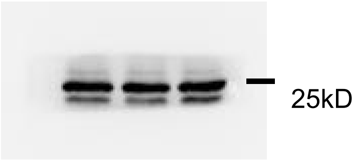

Fig6B
Input： *Myc*

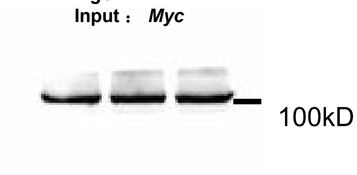

Fig6B
Input： *β-actin*

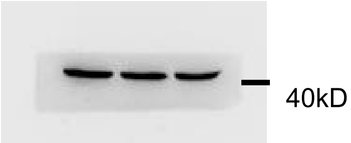

Figure 6C

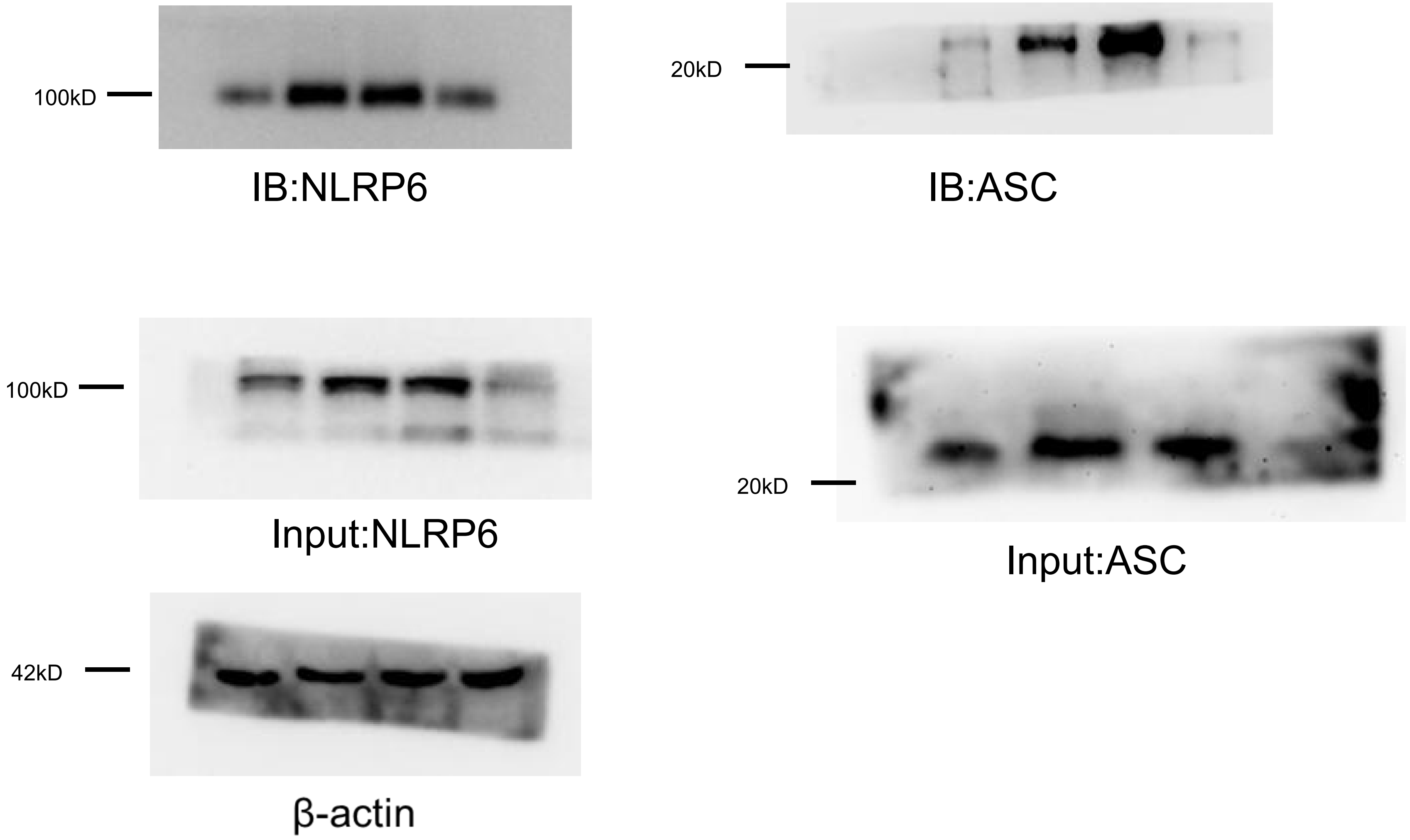
100kD
IB:NLRP6
20kD
IB:ASC
100kD
Input:NLRP6
20kD
Input:ASC
42kD
β-actin
