# Supplementary material for: BRCC3 mediates inflammation and pyroptosis in cerebral ischemia/reperfusion injury by activating the NLRP6 inflammasome
Source: CNS Neurosci Ther. 2024 Mar 28;30(3):e14697. doi: 10.1111/cns.14697 (PMC10973773; doi:10.1111/cns.14697)
Supplement: Supplementary file 1 — Data S1–S2 [file CNS-30-e14697-s001.zip › supplementary materials.pptx]

## Slide 1
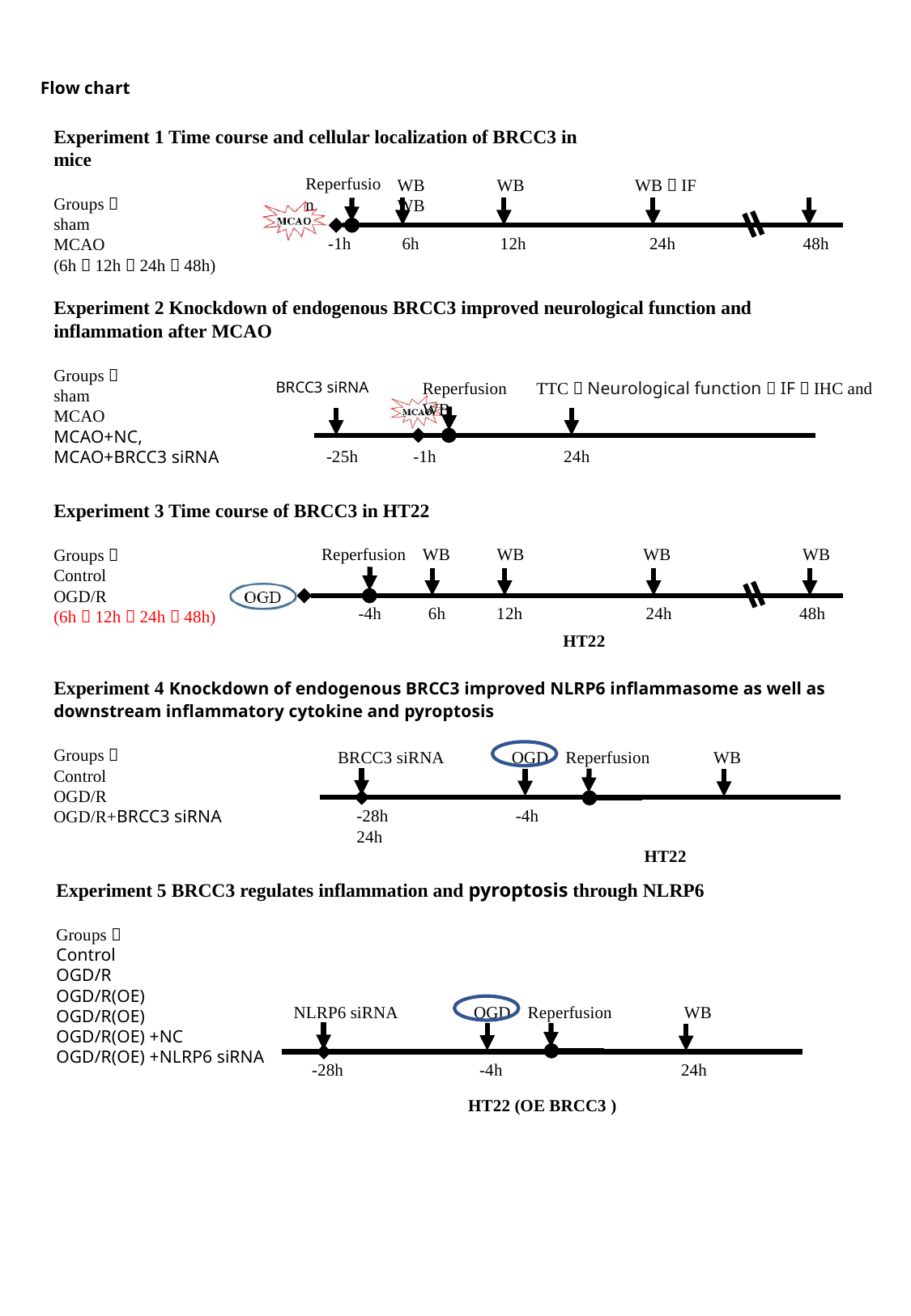

Flow chart
Experiment 1 Time course and cellular localization of BRCC3 in mice
Groups：
sham
MCAO
(6h，12h，24h，48h)
Reperfusion
WB WB WB，IF WB
 -1h 6h 12h 24h 48h
Experiment 2 Knockdown of endogenous BRCC3 improved neurological function and inflammation after MCAO
Groups：
sham
MCAO
MCAO+NC,
MCAO+BRCC3 siRNA
BRCC3 siRNA
Reperfusion TTC，Neurological function，IF，IHC and WB
-25h -1h 24h
Experiment 3 Time course of BRCC3 in HT22
Groups：
Control
OGD/R
(6h，12h，24h，48h)
 Reperfusion WB WB WB WB
 -4h 6h 12h 24h 48h
HT22
Experiment 4 Knockdown of endogenous BRCC3 improved NLRP6 inflammasome as well as downstream inflammatory cytokine and pyroptosis
Groups：
Control
OGD/R
OGD/R+BRCC3 siRNA
BRCC3 siRNA OGD Reperfusion WB
-28h -4h 24h
HT22
Experiment 5 BRCC3 regulates inflammation and pyroptosis through NLRP6
Groups：
Control
OGD/R
OGD/R(OE)
OGD/R(OE)
OGD/R(OE) +NC
OGD/R(OE) +NLRP6 siRNA
NLRP6 siRNA OGD Reperfusion WB
 -28h -4h 24h
HT22 (OE BRCC3 )
