# Supplementary material for: BRCC3 mediates inflammation and pyroptosis in cerebral ischemia/reperfusion injury by activating the NLRP6 inflammasome
Source: CNS Neurosci Ther. 2024 Mar 28;30(3):e14697. doi: 10.1111/cns.14697 (PMC10973773; doi:10.1111/cns.14697)
Supplement: Supplementary file 1 — Data S1–S2 [file CNS-30-e14697-s001.zip › cns14697-sup-0001-supplementary-materials.pdf]

S1

Experiment 1 Time course and cellular localization of BRCC3 in mice

Groups:
sham
MCAO
(6h, 12h, 24h, 48h)

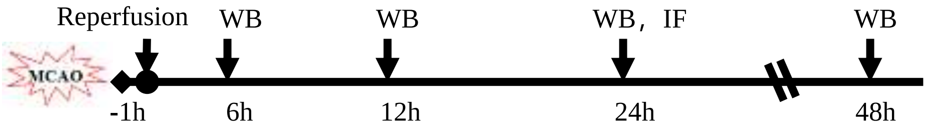

Experiment 2 Knockdown of endogenous BRCC3 improved neurological function and inflammation after MCAO

Groups:
sham
MCAO
MCAO+NC,
MCAO+BRCC3 siRNA

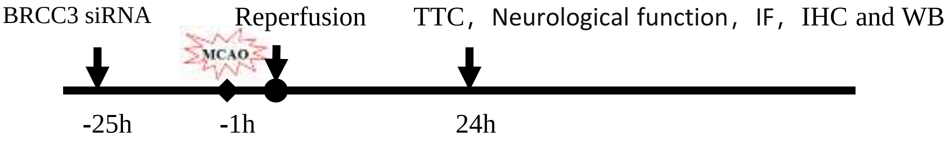

Experiment 3 Time course of BRCC3 in HT22

Groups:
Control
OGD/R
(6h, 12h, 24h, 48h)

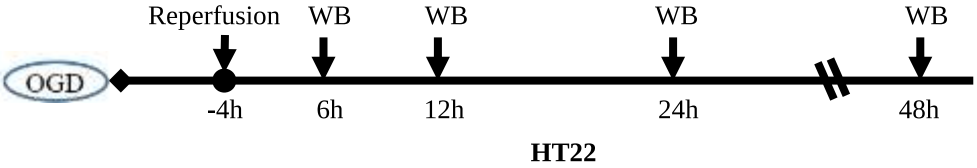

Experiment 4 Knockdown of endogenous BRCC3 improved NLRP6 inflammasome as well as downstream inflammatory cytokine and pyroptosis

Groups:
Control
OGD/R
OGD/R+BRCC3 siRNA

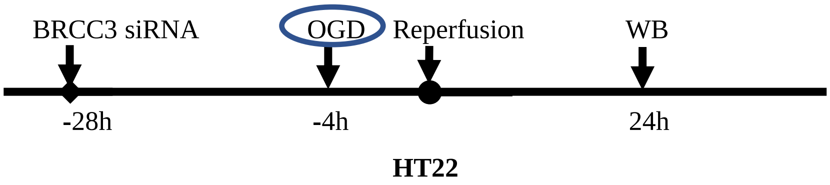

Experiment 5 BRCC3 regulates inflammation and pyroptosis through NLRP6

Groups:
Control (EV)
OGD/R (EV)
OGD/R(OE)
OGD/R(OE) +NC
OGD/R(OE) +NLRP6 siRNA

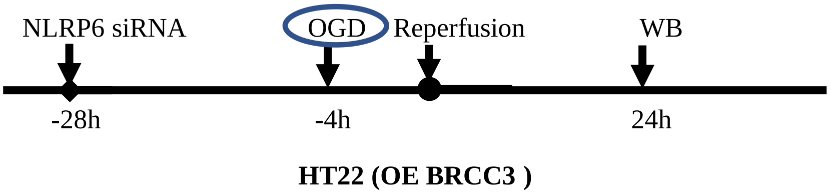

S1 Experimental design and animal groups. BRCC3, BRCA1-BRCA2-containing complex subunit 3; MCAO, middle cerebral artery occlusion; NC, negative Control; HE, hematoxylin–eosin staining; TTC, 2,3,5-triphenyltetrazolium chloride staining; IF, immunofluorescence; WB, western blot; OGD, oxygen-glucose deprivation; siRNA, small interfering RNA; EV , empty vector and OE , overexpression.

S2A

**NLRP6**	**Forward**	**5’-CCGCTCGAGTGATGCCGCTGGAGC-3’**
	**Reverse**	**5’-GCTCTAGATTATTTTGAATATATGATGGACAGATCTGGCTTT-3’**
**BRCC3**	**Forward**	**5-GCTCTAGAATGGCGGTGCAGGTGGTG-3’**
	**Reverse**	**5’-CCGCTCGAGTTATTCTAGGGAAGACAGCTCTTCCA-3’**
**BRCC3-N**	**Forward**	**5’-GGAATTCGCGGTGCAGGTGGTGCAA-3’**
	**Reverse**	**5’-GCTCTAGATTATTTTTGGGCTTGTATGG-3’**
**BRCC3-C**	**Forward**	**5’-GGAATTCGCCCAAAAAAGCTC-3’**
	**Reverse**	**5’-GCTCTAGATTATTCTAGGGAAGACAG-3’**

S2B

**BRCC3-mus**	**Forward**	**5’-GCTCTAGAATGGCGGTGCAGGTGGTG**
	**Reverse**	**5’-CCGCTCGAGTTATTCTAGGGAAGACAGCTCTTCCA**

S2A-B Design primers for plasmid construction .
